# Enhanced photocatalytic degradation of amoxicillin using a spinning disc photocatalytic reactor (SDPR) with a novel Fe_3_O_4_@void@CuO/ZnO yolk-shell thin film nanostructure

**DOI:** 10.1038/s41598-023-43437-8

**Published:** 2023-09-27

**Authors:** Saeid Fallahizadeh, Mitra Gholami, Mahmood Reza Rahimi, Ali Esrafili, Mahdi Farzadkia, Majid Kermani

**Affiliations:** 1 Research Center for Environmental Health Technology, Iran University of Medical Sciences, Tehran, Iran; 2 Department of Environmental Health Engineering, School of Public Health, Iran University of Medical Sciences, Tehran, Iran; 3 Process Intensification Laboratory, Department of Chemical Engineering, Yasouj University, Yasouj, 75918-74831 Iran

**Keywords:** Environmental sciences, Chemistry

## Abstract

Antibiotics are resistant compounds with low biological degradation that generally cannot be removed by conventional wastewater treatment processes. The use of yolk-shell nanostructures in spinning disc photocatalytic reactor (SDPR) enhances the removal efficiency due to their high surface-to-volume ratio and increased interaction between catalyst particles and reactants. The purpose of this study is to investigate the SDPR equipped to Fe_3_O_4_@void@CuO/ZnO yolk-shell thin film nanostructure (FCZ YS) in the presence of visible light illumination in the photocatalytic degradation of amoxicillin (AMX) from aqueous solutions. Stober, co-precipitation, and self-transformation methods were used for the synthesis of FCZ YS thin film nanostructure and the physical and chemical characteristics of the catalyst were analyzed by XRD, VSM,, EDX, FESEM, TEM, AFM, BET, contact angle (CA), and DRS. Then, the effect of different parameters including pH (3–11), initial concentration of AMX (10–50 mg/L), flow rate (10–25 mL/s) and rotational speed (100–400 rpm) at different times in the photocatalytic degradation of AMX were studied. The obtained results indicated that the highest degradation efficiency of 97.6% and constant reaction rate of AMX were obtained under LED visible light illumination and optimal conditions of pH = 5, initial AMX concentration of 30 mg/L, solution flow rate of 15 mL/s, rotational speed of 300 rpm and illumination time of 80 min. The durability and reusability of the nanostructure were tested, that after 5 runs had a suitable degradation rate. Considering the appropriate efficiency of amoxicillin degradation by FCZ YS nanostructure, the use of Fe_3_O_4_@void@CuO/ZnO thin film in SDPR is suggested in water and wastewater treatment processes.

## Introduction

Pharmaceuticals, including antibiotics, are a type of micropollutant that is increasingly being found in the environment. These substances can enter the environment through various sources such as wastewater from pharmaceutical production, improper disposal of medications, runoff from agricultural fields, and other human-related activities^1–3^. The presence of pharmaceuticals in the environment is concerning due to their resistance to degradation. They have been detected in various environmental samples worldwide, including wastewater treatment plant effluents, surface water, seawater, groundwater, soils, and sediments. This persistence is closely related to their bioresistant nature, which means they are not easily broken down by natural processes. Among pharmaceutical compounds, antibiotics have received particular attention because of their potential role in the development of antibiotic-resistant bacteria^4^. Antibiotics are extensively used in human and veterinary medicine, as well as in aquaculture, to prevent or treat microbial infections^5,6^. Many antibiotics have been found to be recalcitrant, meaning they resist degradation, under aerobic conditions typically found in conventional wastewater treatment plants. As a result, these antibiotics can escape largely intact into the environment. To address the issue of antibiotic pollution, non-biological methods have been employed for their treatment^7,8^. These methods include advanced oxidation processes, membrane separation, adsorption, coagulation, and various combinations thereof^9^. Researchers have conducted numerous studies in this field, and comprehensive reviews have summarized the most representative findings. Amoxicillin (AMX) is a semi-synthetic β-lactam antibiotic commonly used in human medicine to treat various diseases. It is also used in veterinary practice as a growth promoter. AMX is excreted from the body with minimal metabolism, so it is likely to be found in environmental samples rather than in the form of metabolites. It has been detected at concentrations in the µg/L range in environmental samples, including secondary treated effluents and surface water. In some cases, the concentration of AMX in antibiotic manufacturing effluents can reach the mg/L level^10^. Overall, the presence of pharmaceuticals, particularly antibiotics, in the environment is a significant concern due to their potential ecological and human health impacts. Efforts are being made to develop and implement effective treatment methods to minimize their release into the environment and mitigate the development of antibiotic resistance^11,12^.

All advanced oxidation processes are based on the production of free radicals with high oxidizing power such as hydroxyl radicals and superoxide radicals^13,14^. Advanced oxidation processes include photocatalytic processes, Fenton process, photofenton process and ozonation process. The use of conventional methods and techniques, such as adsorption or coagulation, transports pollutants to another phase and rarely degrades or removes them^15^. Other conventional methods, such as sedimentation, filtration, and membrane, have high operating costs or produce a hazardous by-product^16^. This has led to the rapid development of advanced oxidation processes. Because of AOP processes, instead of separating or transferring the pollutant to another phase, it directly degrades them and converts them into harmless substances such as carbon dioxide (CO_2_) and water (H_2_O). Therefore, this process is very important from an environmental point of view^17^.

Conventional photocatalysis reactors, which are carried out with powder catalysts in batch systems, suffer from slow reaction kinetics and are costly and difficult to industrialize, principally due to demands for separation equipment to cope with the powder catalyst^18^. In photocatalytic processes, deposited thin film catalysts have become significantly favorite to treat the need for the post-segregation phase needed with powders, howsoever this results in mass transfer limitations due to decreased concentration gradients and surface area of catalyst in the liquid solution^19,20^. Therefore the spinning disc reactor has been pursued as a process intensification technology for the photocatalytic degradation of pollutants in water bodies in order to prevail these problems^21^. In the SDR, the aforementioned limitations can be overcome by coating the catalyst on a substrate with strict adhesion, not reducing the catalyst activity and surface area^22,23^. The convenient light distribution within the reactor and adequate mass transfer between the pollutants and catalysts are the main design criteria in this kind of photocatalytic reactor^24^. These photocatalytic reactors can be established in two forms, the slurry system that the catalyst is suspended in the liquid solution, and the catalyst-immobilized system that includes the deposition of the catalyst on an appropriate substrate^25^. The slurry system needs catalyst segregation and recovery at the finite of the photocatalytic reaction which may increase the operational costs and reduce the mass transfer rate and system efficiency^26,27^. To eliminate these limitations, deposited photocatalytic reactors have been expanded by coating the catalyst onto different substrates such as glass, Teflon, ceramic, zeolite, alumina, and stainless steel^22^. A suitable substrate should have favorable bonding sites and surface, suitable adhesion to the catalyst, excellent photocatalytic activity, superior thermal and mechanical durability, corrosion resistance, and self-cleaning features^27,28^.

Photocatalytic heterogeneous innovation, which belongs to advanced oxidation processes, has emerged as a highly promising approach for the removal of organic pollutants in wastewater. It offers several advantages such as low cost, non-toxicity, low energy requirement, safety, reusability, and the ability to completely degrade organic compounds^29^. Among semiconductor oxides, ZnO is a widely used photocatalyst due to its easy accessibility and affordability^30^. Numerous studies have investigated the photocatalytic activities of ZnO in various forms, including powder and thin film, against different pollutants^31,32^. However, photocatalysts prepared in powder form often suffer from activity losses due to difficulties in separation from the solution medium^33^. To overcome these issues, the thin film structure has been employed, which enhances photocatalytic performance. Nevertheless, it has been reported that losses of photocatalyst occur on the external layer of the thin film during repeated experiments^34^.

Because of its natural abundance as a starting material, low cost production processing, nontoxic makeup, and reasonably good electrical and optical properties, copper oxide (CuO) has been investigated as a p-type semiconductor material with narrow band gap. In many applications, such as catalysis, photocatalysis, humidity and gas sensors, CuO with a lower band gap between 1.2 and 1.9 eV is typically used^35^. Due to the rapid recombination of electron–hole pairs generated by light, the CuO, however, displayed low photocatalytic activity. Effectively reducing the recombination of photogenerated electrons-holes can be achieved by binding CuO to other materials, such as ZnO or TiO_2_^36,37^. Re-collection of NPs from treated water is another issue with CuO NP applications in industry, which drives up costs and taints the water that has been treated. Using magnetic photocatalysts such as Fe_3_O_4_ to solve these issues is a potential solution^38^. Increasingly, the morphology change of the catalyst is a significant manner to progress photocatalytic exclusivity^39^. It has been demonstrated that the fluorescence wavelength, quantum yields and their half-life would be modified by growing a shell of another semiconductor such as Au@TiO_2_, Fe_3_O_4_@Au, etc^40,41^. The hollow nanostructure has been enticed much heed because of its vast specific surface area and plentiful active sites of the catalyst. Alongside, light will be reflected and scattered repeatedly in the hollow internal cavity, so that to decline the propagation interval of electron–hole pairs and uttermost the usage of light^41^. For better progress the photocatalytic efficiency in yolk-shell nanostructure, which involves the core inside a void space cinctured by a semiconductor shell, has been expanded. This nanostructure contains the preferences of both hollow structure and core–shell structure^42,43^.

Spinning disc photocatalytic reactors are a type of photocatalytic reactor used for carrying out photocatalytic reactions. In these reactors, a disc is placed horizontally and rotates rapidly around its axis. A catalyst is placed on the surface of the disc, which initiates chemical reactions using sunlight or other light sources. In these reactors, catalysts (usually nanoparticles) are used as light absorbers and, upon light irradiation, they excite electrons from the valance band to conduction band. These electrons then interact with the molecules present at the reaction site and initiate chemical reactions. Spinning disc photocatalytic reactors offer several advantages, including increased catalyst-light contact area, high efficiency in pollutant removal, and uniform light distribution, making them particularly important^44^.

In this study, we developed and fabricated a visible-light-driven thin-film photocatalyst with a type of yolk-shell nanostructure supported by SDPR as advanced intensification system. The photocatalyst incorporated Fe_3_O_4_@void@CuO/ZnO yolk-shell nanostructure film and was designed specifically to investigate the degradation of amoxicillin (AMX), a representative antibiotic found in aqueous media. The system was equipped with blue LED lamps, which were adjustable in intensity by modifying their distance from the SDPR. This adjustment allowed for compensation of non-uniform light distribution and achieved effective light penetration depth, thereby minimizing the necessary light exposure to activate the catalyst. To deposit the visible light-activated Fe_3_O_4_@void@CuO/ZnO yolk-shell photocatalyst thin film, we utilized the spin coating technique on a spinning ceramic disc substrate. Spin coating involves the application of a liquid precursor solution onto a rotating substrate, resulting in the formation of a uniform thin film. Furthermore, we explored and optimized the effects of various factors including pH, initial AMX concentration, flow rate, and rotational speed. These factors play a crucial role in determining the efficiency of the photocatalytic degradation process. Overall, our study aimed to design and fabrication a novel yolk-shell thin film as a visible-light-driven photocatalyst. This thin film successfully facilitated the AMX degradation as a representative antibiotic in aqueous environments.

## Materials and methods

The SDPR consist of a rotating disc (disc diameter = 20 cm) to form cylindrical on which FCZ YS nanostructure catalyst is deposited. The disc was irradiated from above and the distance between light source (blue LED lamp, light intensity 13 mW/cm^2^) and the disc surface was kept constant in all experiments.

### Chemical reagents

Copper nitrate trihydrate (Cu(NO_3_)_2_.3H_2_O), Zinc nitrate hexahydrate (Zn(NO_3_)_2_.6H_2_O), Ferrous chloride tetrahydrate(FeCl_2_∙4H_2_O), Ferric chloride hexahydrate (FeCl_3_∙6H_2_O), 3-Aminopropyl triethoxysilane (APTES), tetraethyl orthosilicate (TEOS), ammonium hydroxide (NH_3_OH, 25% w/w) and other reagents and solvents were purchased from the Merck company (Darmstadt, Germany, www.merck.de). Amoxicillin (purity 99%) was obtained from Dana pharmaceutical company, Iran. All abovementioned reagent and solutions were used without any further purification. Deionized (DI) water with a resistivity of > 18 MΩ cm was applied in all experiments.

### Set up of spinning disc photocatalytic reactor

A spinning disc photocatalytic reactor (SDPR) was applied for the AMX photodecomposition in aqueous solutions as shown in Fig. S1. The operation mechanism of the spinning disc photocatalytic reactor has been described in previous articles^44,45^. In the SDPR, AMX degradation was performed under visible light irradiation by coating a FCZ YS thin film photocatalyst on a ceramic rotating disc. This photocatalytic reactor was equipped in a glass cylindrical encasement, an amended rotating ceramic disc with a catalyst, and blue LED lamps. The mixture of AMX solution enters the storage tank and is poured onto the disc center by a distributor. The distributor is a nozzle with five holes. Inside the reactor that disc can be rotated to range of 100–400 rpm AMX solution moves exterior through the disc edge due to centrifugal force, the liquid is sprinkled on the rotating disc and thinner solution films and very fine droplets could be formed. This system result in promoting the mass transfer coefficient and significantly declines the reactor size compared to conventional photocatalytic reactor^45^. The blue LED lamp is installed above the fixed encasement to supply the light source. The SDR has a control box system for alignment of the disc rotational speed, switching on/off the blue LED lamp, switching on/off the pump (air and water), motor, and mixer. Under LED visible light irradiation, the photocatalytic degradation procedure was carried out in a spinning disc reactor. The reactor was cylindrical Pyrex glass sealed with aluminum foil to remove extra light. The pollutant concentrations was quantified using UV–Vis spectrophotometer (JASCO, V 730). The pH was adjusted using pH meter type 827 pH Lab-metrohm.

### Synthesis of Fe_3_O_4_@void@CuO/ZnO yolk-shell

#### Synthesis of pure Fe_3_O_4_ magnetics nanoparticles

Pure Fe_3_O_4_ magnetics nanoparticles (MNPs) were synthesized through the co-precipitation method of Fe^3+^ and Fe^2+^ ions (molar ratio 2:1) in alkali solution. FeCl_3_∙6H_2_O (6.1 g) and FeCl_2_∙4H_2_O (2.35 g) were dissolved in 100 mL deionized water and kept at 90 °C for 10 min under continuous magnetic stirring and N_2_ atmosphere (Fig. S2). Following, NH_3_OH (10 mL) was added dropwise and then separated with an external magnet. The obtained Fe_3_O_4_ MNPs were rinsed with distilled water and ethanol for three times to remove impurities and then dried in the oven at 60 °C for 12 h.

#### Synthesis of Fe_3_O_4_@SiO_2_ core–shell nanostructures

The Fe_3_O_4_@SiO_2_ core–shell nanostructures (FS) were synthesized by a modified Stöber method^46^. Firstly, the as-prepared 1 g Fe_3_O_4_ MNPs were dispersed into a mixed solution of 100 mL of methanol and 16 mL of deionized water by ultrasonic for 30 min. Next, in a round bottom flask 2 necks, 5 mL ammonia hydroxide solution (25%) and 3 mL TEOS were added into the above mixture solution and stirred for 24 h. Then, the final products after separation by external magnet were washed with deionized water and HCl (0.1 M) for three times, and dried in an oven at 70 °C for 8 h (Fig. S3).

#### Fe_3_O_4_@SiO_2_ core–shell nanostructures surface modification

A solution of the Fe_3_O_4_@SiO_2_-NH_2_ (FSN) nanostructures was prepared by mixing 2 g Fe_3_O_4_@SiO_2_ core–shell NSs in 80 mL of methanol and stirred by ultrasonic for 15 min. Then 5.3 mL APTES was added to mixed solution and under magnetic stirrer and reflux at 65 °C for 24 h until the reaction was complete. The resultant product after separation by external magnet was dried in an oven at 70 °C for 10 h (Fig. S4).

#### Synthesis of Fe_3_O_4_@SiO_2_-NH_2_@CuO/ZnO core–shell nanostructures

The Fe_3_O_4_@SiO_2_-NH_2_@CuO/ZnO core–shell (FSCZ) composite were synthesized using a hydrothermal process. In this three-step method, first, 1.115 g Zn(NO_3_)_2_.6H_2_O and 1.8 g Cu(NO_3_)_2_·3H_2_O were dissolved in 75 mL deionized water. Second, 0.5 g Fe_3_O_4_@SiO_2_-NH_2_ core–shell nanostructures was dissolved in 50 mL deionized water. Then, 15 mL NH_3_OH were added drop wise to the mixed solution of the previous two steps and kept under a magnetic stirrer for 24 h. Finally, after separation by external magnet and washing several times with distilled water and ethanol, the resulting product was dried in an oven at 60 °C for 12 h (Fig. S5).

#### Synthesis of Fe_3_O_4_@void@CuO/ZnO yolk-shell nanostructures

The Fe_3_O_4_@void@CuO/ZnO yolk-shell (FCZ YS) composite were synthesized using a self-transformation process (Fig. S6). Briefly, 1 g of the as-synthesized Fe_3_O_4_@SiO_2_-NH_2_@CuO/ZnO core–shell NSs were dispersed in 50 mL deionized water by ultrasonication for 15 min and then was kept at 70 °C for 48 h until the SiO_2_ shell is removed and to form the yolk-shell structure through a self-transformation process^47^. Afterward, the yolk-shell structure were collected by magnet and washed with ethanol and DI water and dried at 60 °C for 24 h thoroughly in vacuum oven.

#### Deposition of Fe_3_O_4_@void@CuO/ZnO yolk-shell NSs thin film on the disc

The Fe_3_O_4_@void@CuO/ZnO yolk-shell thin film was deposited on ceramic disc substrate by sol–gel spin coating method (Fig. S7). Prior to film deposition, the ceramic disc substrate was cleaned and washed thoroughly with acetone, ethanol solutions and DI water and then dried for any contamination and precipitation removal at 100 °C for 1 h. At first, 1 wt% polyvinyl alcohol (PVA) in 25 mL DI water was placed in a sealed container for 3 h at 80 °C (to promote complete swelling) in ultrasonic bath to access a transparent solution. Afterward, to achieve a homogenous solution 40 g/L of as synthesized Fe_3_O_4_@void@CuO/ZnO yolk-shell NS was dissolved in 25 ml DI water. The mixture of Fe_3_O_4_@void@CuO/ZnO yolk-shell NS and PVA solutions was subjected to ultrasonication for 1 h until a homogeneous solution was obtained. The new solution obtained was used for the deposition of a ceramic disc by spin coating at a speed of 1000 rpm for 60 s. In order to reach the desired deposition, five layers were placed on the disc and dried at 60 °C for 15 min after each coating, and finally at 250 °C for 1 h in a vacuum oven.

## Results and discussion

### Characterization of nanostructured photocatalysts

The nanostructure photocatalysts were characterized by Field Emission Scanning Electron Microscope (FE-SEM TESCAN MIRA3 and FE-SEM Zeiss-Sigma 300), Energy Dispersive X-ray Spectroscopy (EDX), Transmission Electron Microscopy (TEM, Philips EM 208S), X-ray diffraction (XRD, Rigaku Ultima IV, Japan), UV–Visible Spectrophotometer (JASCO V-730, Japan), and Vibrating Sample Magnetometer (VSM, MDK VSM, Iran). Atomic Force Microscope (AFM Nanowizard 2, JPK company, Germany), Diffuse Reflectance Spectroscopy (DRS S-4100 SCINCO), Contact angle (Contact angle CAG-20), and BET (BELSORP mini II, Japan).

#### XRD analysis

**Figure 1:**
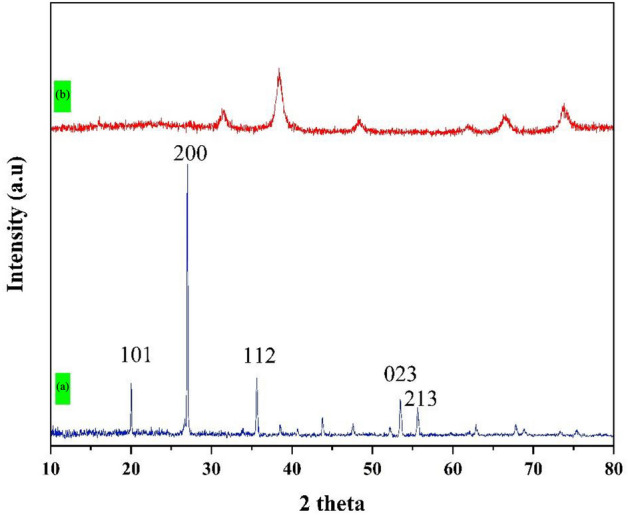
The XRD spectra of pristine disc (**a**) and Fe_3_O_4_@void@CuO/ZnO yolk-shell film (**b**).

XRD patterns were prepared using a Rigaku Ultima IV apparatus with Cu Ka radiation (wavelength, l = 1.5418 Å). X-ray diffraction analysis is noticed as a favorable method in the recognition of a crystal structure and crystallographic pattern of as-prepared samples. Figure S8a–c represents the XRD patterns of Fe_3_O_4_, Fe_3_O_4_@SiO_2_ core–shell nanostructure, and Fe_3_O_4_@SiO_2_@CuO/ZnO core–shell nanostructure. As illustrated in Fig. S8-a, an good agreement between the XRD pattern of Fe_3_O_4_ MNPs in the present study and those recorded in JCPDS No: 088-0315, peak list: 30.16° (220), 35.52° (311), 43.17° (400), 53.56° (422), 57.1° (511) and 62.7° (440) was found^48^. Impurity peak is not observed. Compared with Fig. 1a, the coating of SiO_2_ layer brings slight decrease in intensity to the XRD pattern of Fe_3_O_4_@SiO_2_ core–shell (Fig. S8-b) that can be cause the existence of amorphous silica. After coating zinc oxide (ZnO) and copper oxide (CuO) grains on the surface of the Fe_3_O_4_@SiO_2_ core–shell nanostrucure (Fig. S8-c), several new peaks appear at 2θ 31.77, 34.42, 36.25, 47,54, 56.6, 62.86 and 67.96 which can be indexed to the ZnO hexagonal wurtzite phase (JCPDS card No. 36-1451) and at 2θ 32.51, 35.45, 55.35, 38.73, 38.92, 48.76, 61.57 and 68.14 which can be indexed (110), (002), (− 111), (111), (200), (− 202), (− 113) and (220) to the monoclinic copper oxide(JCPDS card No. 005-0661), respectively.

The crystal structure of pristine disc (PD) and Fe_3_O_4_@void@CuO/ZnO photocatalytic film was investigated using XRD analysis (Fig. 1). The XRD pattern in Fig. 1a of the important peaks of the ceramic substrate at 2θ including zirconium with crystal planes (101), (002), (112), (211) and (213), titanium dioxide with crystal planes (400), (002), (501) and (402) and sillimanite with crystal planes (141), (320) and (324) is attributed. The peaks formed in coating the photocatalyst film on the ceramic substrate are the same as the peaks in the Fig. S8-c, which confirms the proper film of the yolk-shell structure on the ceramic substrate.

#### VSM analysis

**Figure 2:**
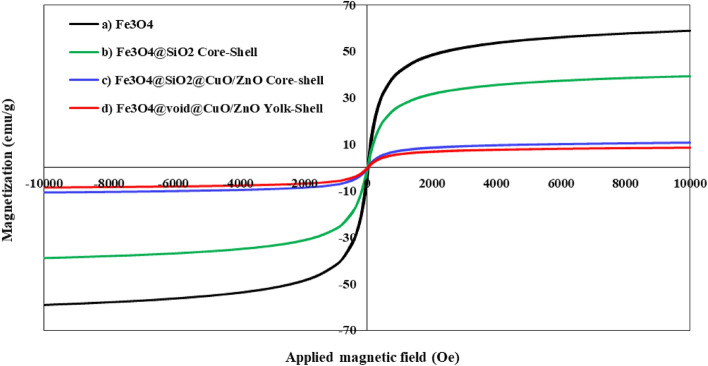
Magnetic saturation of synthesized samples at room temperature of (**a**) Fe_3_O_4_, (**b**) Fe_3_O_4_@SiO_2_, (**c**) Fe_3_O_4_@SiO_2_@CuO/ZnO Core–Shell and (**d**) Fe_3_O_4_@void@CuO/ZnO Yolk-Shell.

VSM pattern of pure Fe_3_O_4_, Fe_3_O_4_@SiO_2_, Fe_3_O_4_@SiO_2_@CuO/ZnO core–shell and Fe_3_O_4_@void@CuO/ZnO yolk-shell nanostructure are examined in Fig. 2 at room temperature under an external magnetic field from − 10,000 to 10,000 Oersted (Oe). Magnetic saturation values of 58.92 emu/g for pure Fe_3_O_4_, 39.34 emu/g for Fe_3_O_4_@SiO_2_ were shown, which in both samples show paramagnetic properties for optimal separation (Fig. 2a, b)^49^. The decrease in the magnetic properties of Fe_3_O_4_@SiO_2_ in Fig. 2b compared to pure Fe_3_O_4_ can be due to the coating of the magnetic Fe_3_O_4_ core by the non-magnetic coating TEOS (SiO_2_) as a shell^50^. These results showed that Fe_3_O_4_ magnetic nanoparticles were significantly reduced by adding SiO_2_ and amino propyl groups (APTES). The reason is mainly attributed to the presence of non-magnetic materials on the surface of nanoparticles^51^. Also, in Fig. 2c where Fe_3_O_4_@SiO_2_@CuO/ZnO core–shell and Fe_3_O_4_@void@CuO/ZnO yolk-shell samples were examined, the results showed that the magnetic behavior of Fe_3_O_4_@SiO_2_@CuO/ZnO core–shell nanostructure decreases with the addition of CuO and ZnO to Fe_3_O_4_@SiO_2_ core–shell was 10.77 emu/g. Although the magnetic saturation has decreased from 58.92 emu/g for Fe_3_O_4_ to 10.77 for Fe_3_O_4_@SiO_2_@CuO/ZnO core–shell nanostructure, but the synthesized core–shell nanostructure still maintain their magnetic properties and behavior. The saturation magnetization for Fe_3_O_4_@void@CuO-ZnO yolk-shell nanostructure is about 8.61 emu/g, which could be easily separated from the solution by making use of an external magnetic field (Fig. 2d)^52,53^.

#### FE-SEM analysis

FESEM is one of the ways to investigate the nanostructure of a material, including its appearance, surface structure, particle size, and the presence of impurities. Figure S9 shows the FESEM images and EDX of Fe_3_O_4_@SiO_2_ magnetic core–shell nanostructure, Fe_3_O_4_@SiO_2_-NH_2_, Fe_3_O_4_@SiO_2_-NH_2_@CuO-ZnO core–shell and Fe_3_O_4_@void@CuO-ZnO yolk-shell. All the images show that the shape of the particles is almost spherical and has nanometer dimensions. According to Fig. S9-a, the increase in particle size by 76.36 nm compared to the size of Fe_3_O_4_@SiO_2_ and particles modified with TEOS indicates the presence of a layer on Fe_3_O_4_ MNPs. The average particle size in the Fe_3_O_4_@SiO_2_-NH_2_ core–shell nanostructure (FSN) is 81.84 nm, which is due to the addition of an organic amine coating (APTES) on the Fe_3_O_4_@SiO_2_ core–shell nanostructure and for this reason, the size of the particles has become larger compared to the Fe_3_O_4_@SiO_2_ nanostructure (Fig. S9-c^51^. Also, Fig. S9-e and g show Fe_3_O_4_@SiO_2_-NH_2_@CuO-ZnO core–shell and Fe_3_O_4_@CuO-ZnO yolk-shell nanostructures. The results showed that the particle size was 103.22 nm in the core–shell structure and 81.36 nm in the yolk-shell structure. The reason for the reduction in the size of the yolk-shell structure is due to the removal of the SiO_2_ layer in core–shell structure.

The energy-dispersive X-ray spectroscopy (EDX) technique is indeed a convenient method for detecting elements and their weight or atomic percentages in a material. The presence of Fe, Si, and O signals in the EDX analysis indicates that Fe_3_O_4_ nanoparticles are loaded into silica (SiO_2_). This suggests that the Fe_3_O_4_ nanoparticles are embedded within the silica matrix (Fig. S9-b). Regarding other nanostructures, the constituent elements are known in Fig. S9d, f and h. The TEM image in Fig. S9-i depicts that CuO and ZnO particles were stoutly linked and distributed on the external surface of the shell without detriment to the yolk-shell structure.

**Figure 3:**
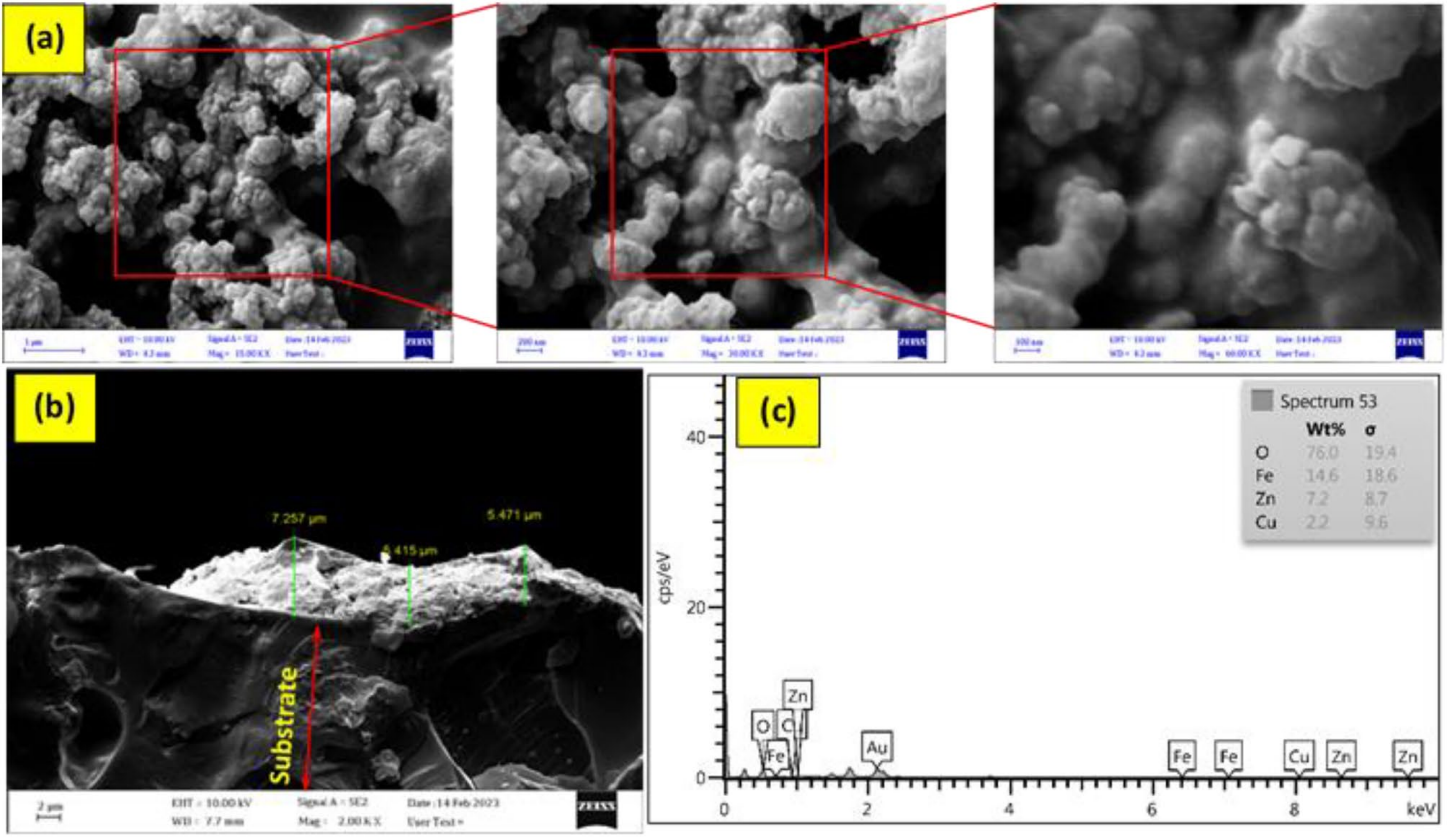
FESEM image of PD-FCZ YS film (**a**) and cross-sectional view of photocatalytic film (**b**) and EDX image (**c**).

Figure 3 shows the results of the cross-sectional and top of view FE-SEM analyses of the FCZ YS NS film on the surface of the ceramic disc. The FE-SEM images shown in Fig. 3a, was confirmed that the FCZ YS NS consists of spherical structures of 74.67 nm in average size on the disc surface. From the Fig. 3b. it has been found that the average thickness of the FCZ YS NS film was about 6 μm. Figure 3c depicts the EDX spectra for deposited thin film in the disc with quantitative elemental distribution. This result depicts that the PD-FCZ YS nanostructure film enhanced the mass transfer rate and initial adsorption of AMX, which result in the photocatalytic activity.

#### Optical analysis

**Figure 4:**
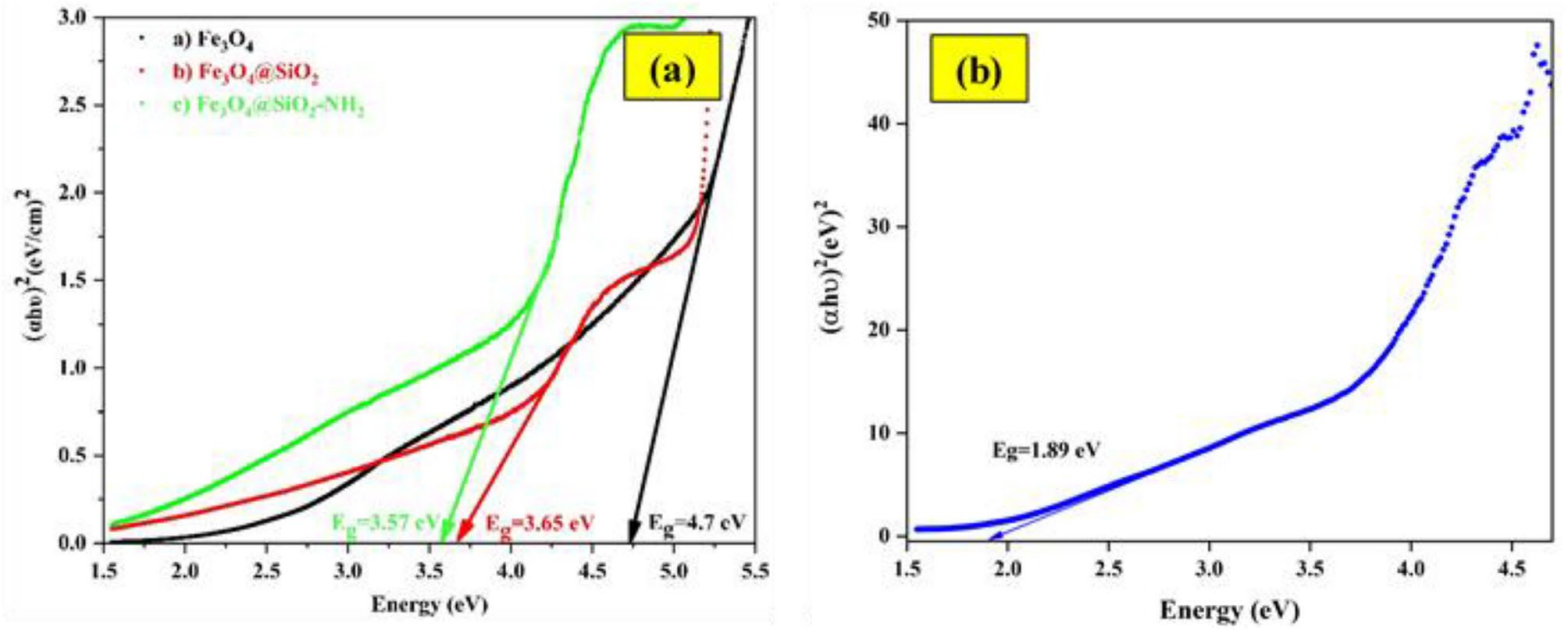
DRS spectra (**a**) Fe_3_O_4_, Fe_3_O_4_@SiO_2_, Fe_3_O_4_@SiO_2_-NH_2_ Core–Shell and (**b**) Fe_3_O_4_@void@CuO/ZnO Yolk-Shell film.

The UV–visible diffuse reflectance spectra (DRS) patterns was used to characterize the optical characteristics of the nanostructures of Fe_3_O_4_, Fe_3_O_4_@SiO_2_, Fe_3_O_4_@SiO_2_-NH_2_ Core–Shell and Fe_3_O_4_@void@CuO/ZnO Yolk-Shell film. Figure 4 depicts that the absorption peaks of the prepared nanostructures were observed within the average UV–Vis absorption terrain [5, 17]. As Fig. 4 shows, the band gap energy (E_g_) for magnetic nanoparticles of Fe_3_O_4_, Fe_3_O_4_@SiO_2_, and Fe_3_O_4_@SiO_2_-NH_2_ are 4.7 eV, 3.65 eV and 3.57 eV, respectively. Compared to the Fe_3_O_4_@SiO_2_-NH_2_ Core–Shell sample, the Fe_3_O_4_@void@CuO/ZnO Yolk-Shell film sample show enhanced absorption in the visible light region (1.89 eV). The reason for this is, on the one hand, due to the increase in the concentration of CuO in the synthesized sample, because CuO is in the visible light region, and on the other hand, the size of the particles can be described according to the FESEM images^38^. This means that as the size of the particles decreases to nano dimensions, their energy levels change from continuous to discrete. Because in normal materials, energy band band are continuous, but in nanomaterials, these bands are discrete. The smaller the size of the nanoparticles, the larger the band gap, and the larger the size of the nanoparticles, the smaller the band gap. Energy band gap increases with decrease in size of the nanoparticles due to electron restriction at nano-size so called “quantum size effect”. Therefore, the change in the band gap is caused by the quantum size effect and the surface effect of Fe_3_O_4_, Fe_3_O_4_@SiO_2_-NH_2_ and Fe_3_O_4_@void@CuO/ZnO yolk-shell nanostructures^54–57^.

#### AFM and contact angle analysis

**Figure 5:**
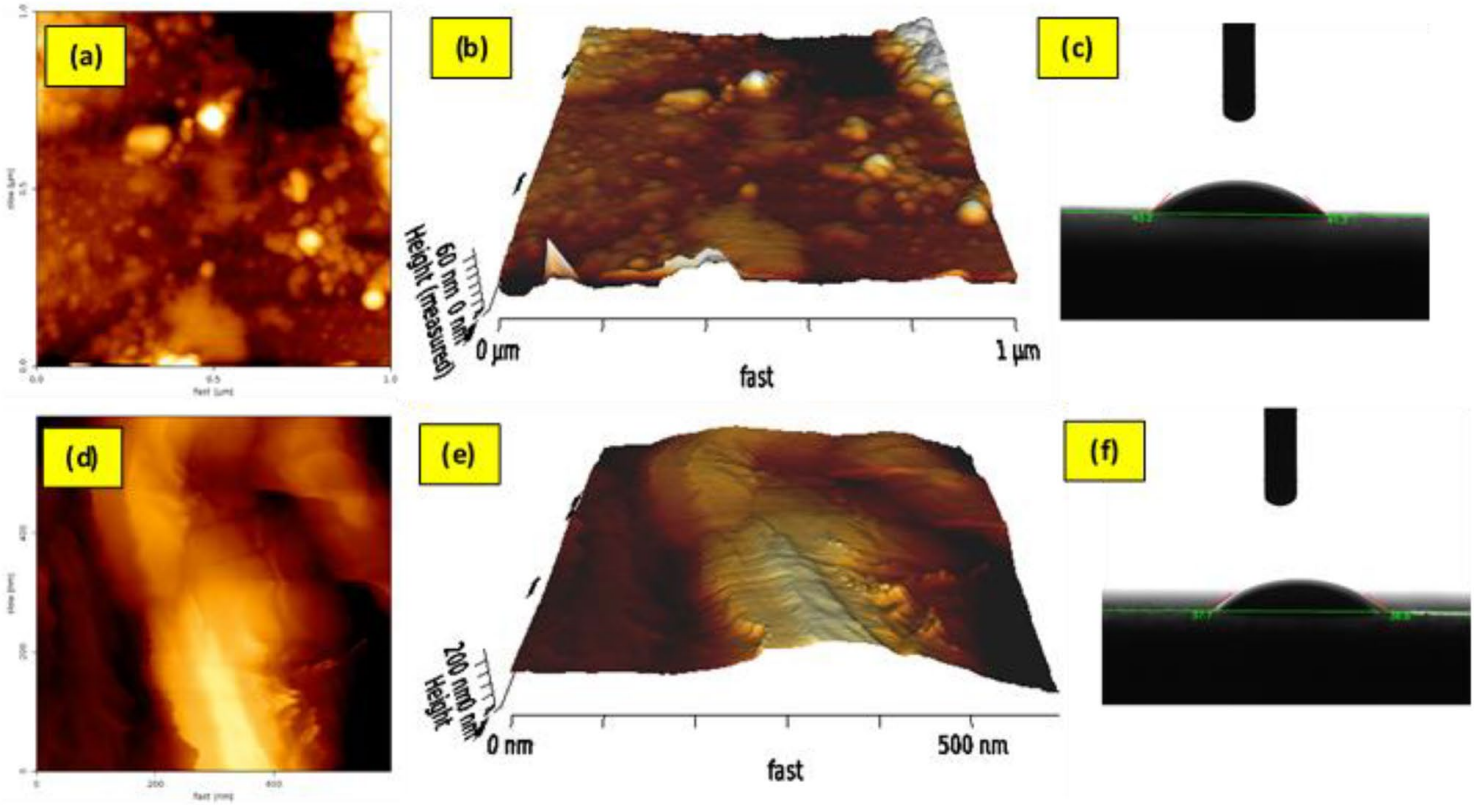
2D and 3D AFM images of (**a**, **b**) PD and (**d**, **e**) PD-FCZ YS film and contact angle images of (**c**) PD and (**f**) PD-FCZ YS film.

Figure 5 AFM 2D and 3D images of pristine disc and FCZ YS deposited on a disc substrate. The AFM images of pristine disc and FCZ yolk-shell NS films are shown in Fig. 5a, b and d, e. AFM analysis has been used to describe the roughness of the substrate and the photocatalytic film coated on the ceramic disc. As shown in the figure, the substrate of the ceramic disc shows a relatively rough surface (average roughness 18.76 nm), while the photocatalytic film shows a higher surface roughness (average roughness 38.4 nm). This increase in surface roughness can be attributed to the presence of a thin film of FCZ yolk-shell nanostructure on the surface of the ceramic disc. The rough surface of the photocatalyst observed in the AFM image was similar to that of the FESEM, indicating the growth of the FCZ yolk-shell nanostructure catalytic film on the substrate surface of the ceramic disc.

One of the important features of the surface is wettability. The Wettability behavior of a surface can be determined by the measurement of the contact angle. The hydrophilicity and hydrophobicity of the immobilized surfaces by FCZ YS photocatalytic film were appraised by water contact angle measurement. The result showed that the hydrophilicity of FCZ YS photocatalytic film is satisfactory (Fig. 5c and f). However, the contact angle reduced upon the formation of FCZ YS photocatalysts film on the ceramic disc surface, enhancing the wettability of the coated disc substrate. An increase in hydrophilicity improved physical features and surface energy to reform the AMX adsorption as an initial monolayer.

#### BET analysis

**Figure 6:**
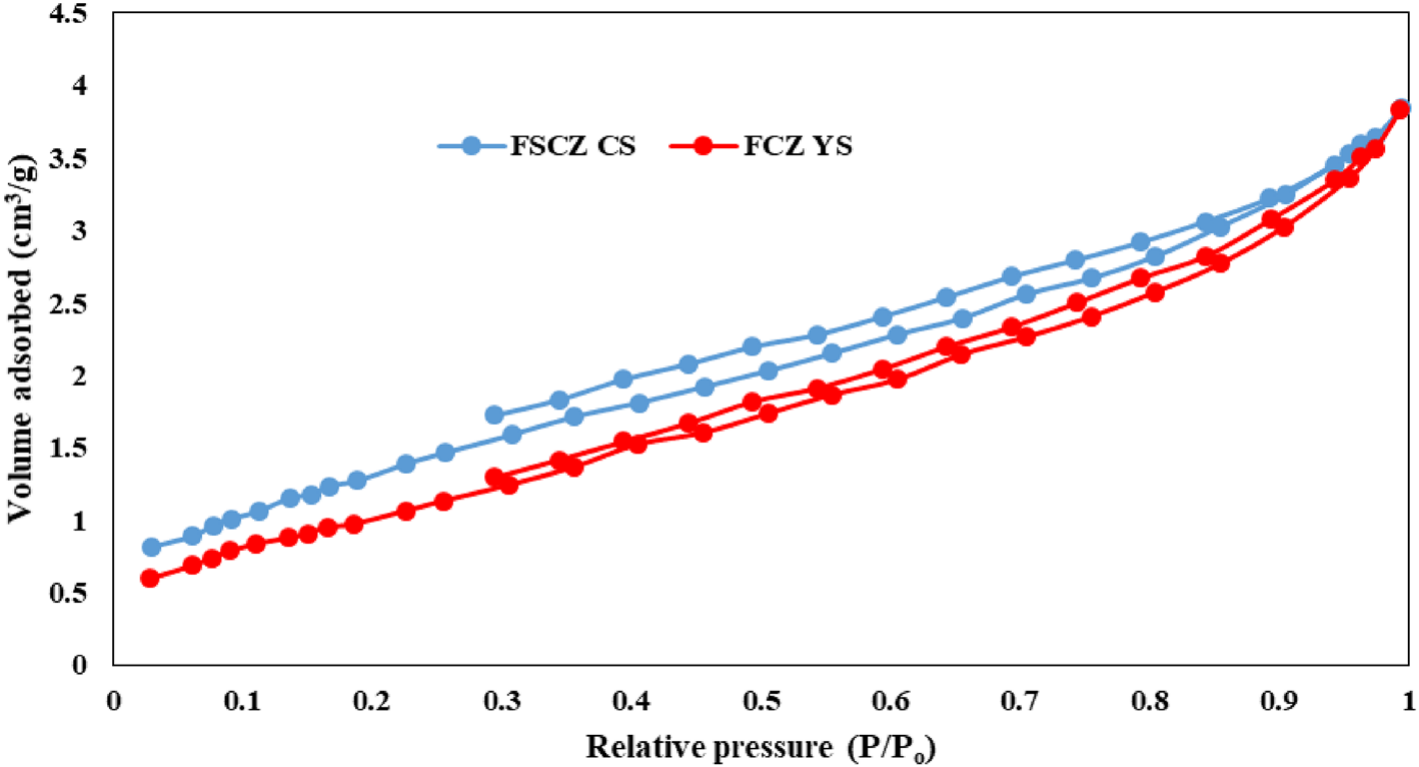
N_2_ adsorption–desorption isotherms of FSCZ CS and FCZ YS.

The BET technique is a commonly used approach to describe the characteristics of porous materials with diameters ranging from 2-50 nm (meso) and 2 nm (micro)^58^. Nitrogen adsorption–desorption isotherms are depicted in Fig. 6. This figure displays that FSCZ CS NSs and FCZ YS NSs exhibits type IV isotherm and 3H hysteresis loop. The BET surface area of FCZ YS Ns is 62.48 m^2^/g, which is remarkably increased compared with 18.98 m^2^/g for the FSCZ CS Ns. FCZ yolk-shell nanostructure showed excellent adsorption and desorption capacity compared to the FSCZ core–shell, and according to the BET specific surface area in the FCZ yolk-shell, it was 3.29 times higher than the FSCZ core–shell nanostructure. Therefore, the number of active sites in FCZ YS Ns has been considerably enhanced and the reaction activity to deal with amoxicillin is increased considerably. The ratio of specific surface area of yolk-shell structure to core–shell (YS to CS ratio) was obtained by Li et al.^59^ 0.95 and Shi et al.^53^ 1.31. Table S1 shows the BET specific surface area in different core–shell and yolk-shell composites and specific surface area ratio of YS to CS. Also, the properties of porosity and specific surface area in the two mentioned structures are listed in Table S2.

### Effect of parameters on photocatalytic degradation

In the process of photocatalytic degradation of AMX by FCZ YS thin film, four factors were optimized of pH, initial pollutant concentration, flow rate and disc rotation speed, respectively. Then, under optimal conditions, the effect of scavengers, mineralization (COD and TOC), reusability and durability of the thin film coated on the disc and comparing FCZ YS and FSCZ CS thin films were investigated.

#### Effect of pH

**Figure 7:**
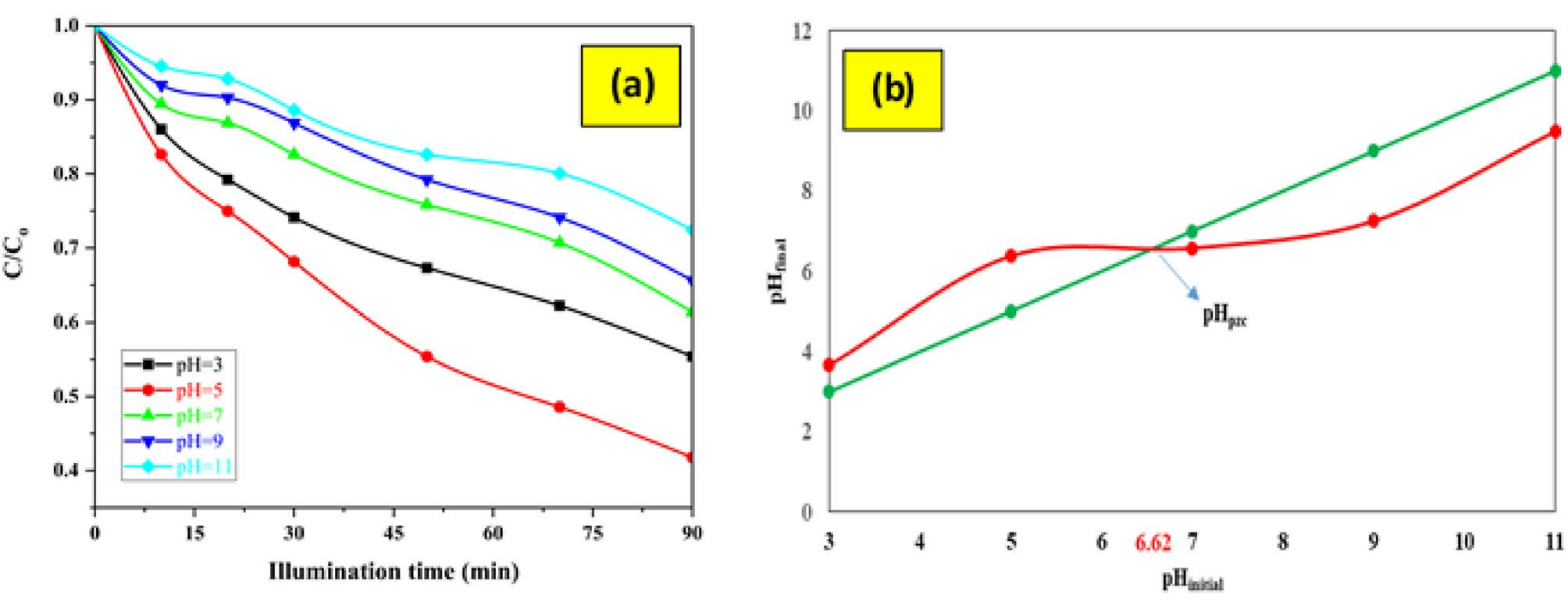
The effect of different solution pH on the photocatalytic degradation of AMX (initial AMX concentration of 50 mg/L, flow rate of 10 mL/s, rotational speed of 200 rpm and illumination time of 90 min) (**a**) and pH_zpc_ plot (**b**).

The pH of the solution plays an important role in the photocatalytic degradation processes of AMX, which is caused by the surface charge of the photocatalyst, the degree of ionization of AMX in the solution, and also the separation of functional groups in the photocatalyst sites. The AMX degradation efficiency is investigated in the pH range of 3–11, an initial AMX concentration of 50 mg/L, flow rate of 10 mL/s, disc rotational speed of 200 rpm and an illumination time of 90 min. The reaction under LED visible light illumination is studied using FCZ YS thin film. Figure 7 plots the degradation of AMX by photodegradation as a function of pH in the range of 3–11. The data achieved using LED visible light are depicted in the Fig. 7a for more comparison. As can be observed in Fig. 7a, the AMX degradation efficiency raises from pH 3 to pH 5, and then decreases from pH 5 to pH 11. In this research, pH 5 was distinguished as the optimum pH value, whereby the FCZ YS thin film photocatalyst has a maximum efficiency of 58.21% at pH 5. AMX and photocatalyst have different performance efficiency at different pH values due to different properties. The pH zero point charge (pH_pzc_) was determined using past studies^60,61^. As the pH_pzc_ for the FCZ YS photocatalyst corresponds to 6.62, as observed in Fig. 7b, the photocatalyst surface is positive at pH < pH_pzc_ and negative at pH > pH_pzc_. The removal efficiency decreases when the pH value goes above 5, reaching the lowest removal rate of 27.57% at pH 11. In Fig. 7b, it is shown that the surface of the yolk-shell nanostructure becomes negatively charged at pH values higher than the point of zero charge (pH_zpc_). This negative charge accumulation on the adsorbent's surface results in electrostatic repulsion between the catalyst and AMX. However, when the pH values are below the pH_zpc_, the catalyst surface carries a positive electrical charge. Since AMX is an anionic compound, there is an electrostatic attraction force between the yolk-shell nanostructure and AMX, causing AMX to degrade on the catalyst surface in an acidic environment. Therefore, pH 5 was chosen as the optimal pH value for the experiments, and all subsequent experiments were conducted at this pH value^7,62^.

#### Effect of initial AMX concentration

**Figure 8:**
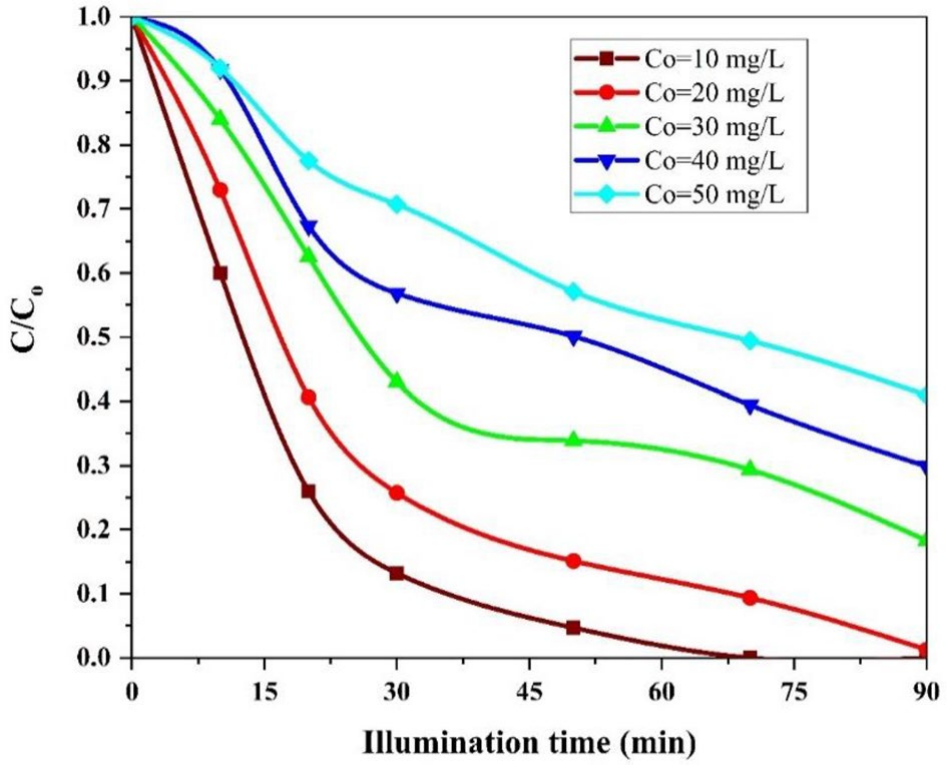
The effect of different initial concentration on the photocatalytic degradation of AMX under conditions: pH = 5, flow rate = 10 mL/s, rotational speed = 200 rpm and illumination time = 90 min.

The effect of the initial concentration of AMX was investigated into 10, 20, 30, 40, and 50 mg/L AMX solutions, respectively, under conditions of optimal pH = 5, flow rate of 10 mL/s, and disc rotation speed of 200 rpm during 90 min. As depicted in Fig. 8. highest AMX removal efficiency were observed at the initial concentrations of 10 mg/L (100%) and 20 mg/L (98.71%) at illumination times of 70 and 90 min, respectively. Then the degradation rate reduces with an increase in the initial AMX concentration from 20 to 50 mg/L. AMX degradation efficiency was optimized at 81.7% (30 mg/L), but it decreased gradually by enhancing the initial AMX concentration of 10 and 50 mg/L with degradation efficiency of 100–59.1%, respectively.

Higher degradation efficiency was obtained for AMX solution with lower starting concentrations. Because due to the surface area of the FCZ YS photocatalyst and the supply of oxidant due to the lower total number of AMX molecules, the degradation will be take place faster^63^. In the intensity of LED visible light radiation, the decrease in degradation efficiency with increasing concentration of AMX solution may be attributed to the competition between AMX molecules, the inhibition of intermediate compounds produced during the photodegradation process, and the decrease in the intensity of light reaching the surface of the FCZ YS catalyst. As Fig. 8 shows, at concentrations higher than 20 mg/L, more light was scattered by the AMX solution and fewer photons are able to reach the FCZ YS catalyst surface, which will largely result in the reduction of electron–hole pair production. As a result, the degradation efficiency decreases with increasing concentration from 20 to 50 mg/L. Since the degradation efficiency of AMX in concentrations of 10 and 20 mg/L are close to each other. Therefore, to optimize the factors of flow rate and disc rotation speed, the initial concentration of 30 mg/L was investigated. When the AMX concentration increases, more molecules are absorbed on the surface of the photocatalyst, so the need for active oxidant species such as hydroxyl radicals and superoxide increases in order to decompose this high concentration of AMX absorbed on the photocatalyst surface^57,64^. Also, the excess concentration absorbed on the surface of the catalyst prevents the absorption of light photons on the surface of the catalyst, as a result, the available active species are not enough to decompose the pollutant with high concentrations, and their deficiency increases with the increase of the pollutant concentration, and many organic compounds leave the reactor without being decomposed. On the other hand, light photons may be absorbed by the organic compounds adsorbed on the surface of the catalyst before they reach the surface of the photocatalyst and prevent access to the surface of the photocatalyst. In addition, during the photocatalytic oxidation process, intermediate compounds are produced that reduce the rate of mineralization or that are placed on the active sites of the catalyst surface and hinder the progress of the photocatalytic process^65^.

#### Effect of flow rate

**Figure 9:**
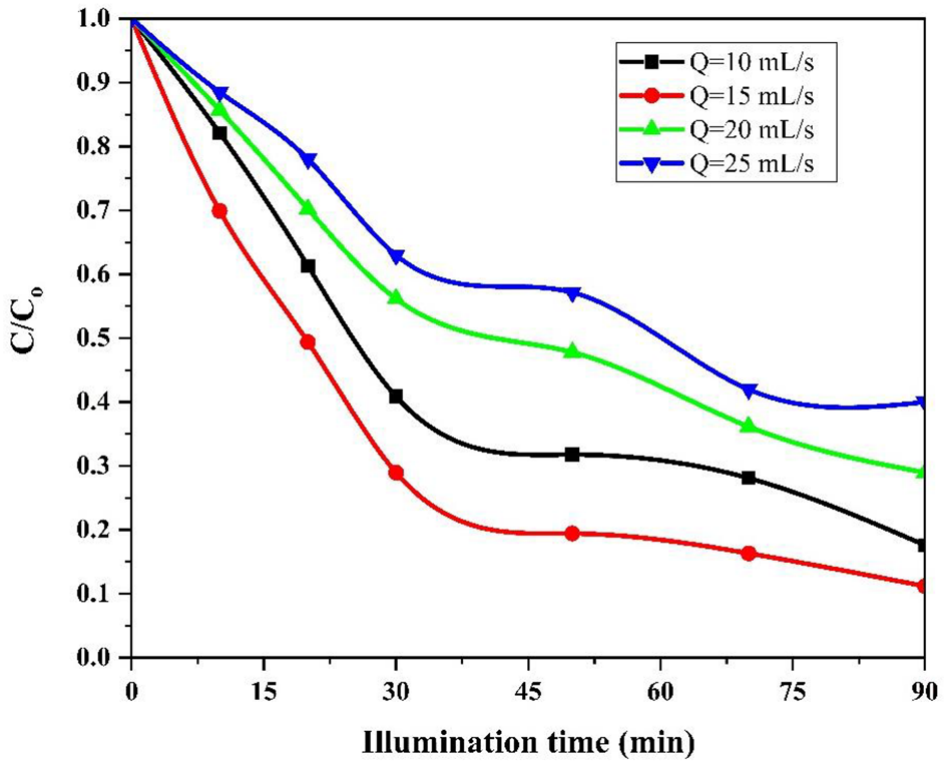
The effect of different flow rate on the photocatalytic degradation of AMX under conditions: pH = 5, initial AMX concentration = 30 mg/L, rotational speed = 200 rpm and illumination time = 90 min.

The effect of solution flow rate on the degradation efficiency of AMX was studied under the conditions of pH = 5, initial AMX concentration of 30 mg/L, rotational speed of 200 rpm and illumination time of 90 min as the coating catalyst on the ceramic disc. The experimental results are depicted in Fig. 8. It can be seen from Fig. 8 that the degradation efficiency of AMX increases with the increasing of flow rate from 10 to 15 mL/s and then decreases from 15 to 25 mL/s. When the flow rate was 10 mL/s, the degradation efficiency was 82.4% after illumination time of 90 min. Therefore, when the flow rate increased to 25 mL/s, the degradation efficiency reached 56%. Three potential reasons are effective in reducing and increasing the flow rate. According to Fig. 9, at a flow rate of 10 mL/s, there is a possibility of breaking down the liquid film and improper distribution of the liquid flow on the disc, as a result, the mixing ability of the liquid film reduces and the decomposition efficiency decreases (at lower flow rate)^66^. In such cases, by increasing the flow rate from 10 to 15 mL/s, the interface between solid–liquid (the FCZ YS catalyst particles and amoxicillin molecules) increased, thus increasing the mass transfer rate and increasing the degradation efficiency (In favorable flow rate). Another reason for the decrease in degradation efficiency with the increase in flow rate from 15 to 25 mL/s can be attributed to the decrease in the residence time of amoxicillin molecules on the disc, which has reduced the exposure time of FCZ YS catalyst to light. On the other hand, with the increase in the flow rate, thicker films of liquid are formed on the disc, which has led to the reduction of the solid–liquid interface and ultimately the efficiency of degradation. Also, with the thickening of the liquid film, the depth of light penetration and the activity of the catalyst have decreased, for this reason, the degradation efficiency has decreased to 56% at the flow rate of 25 mL/s (at higher flow rate)^67^.

#### Effect of rotational speed

**Figure 10:**
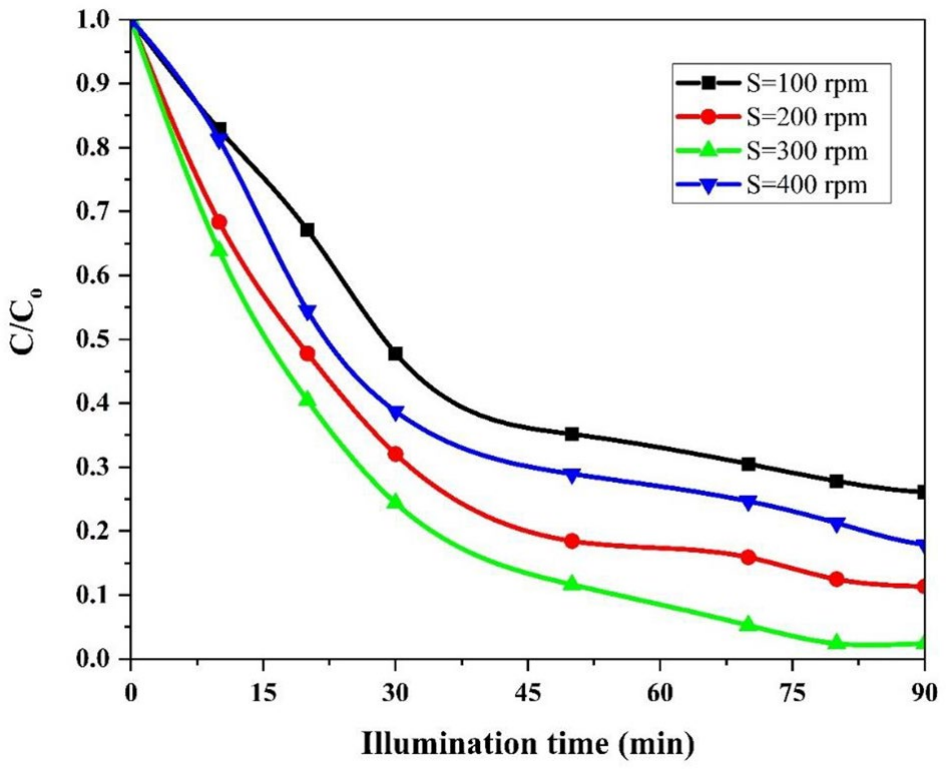
The effect of different rotational speed on the photocatalytic degradation of AMX under conditions: pH = 5, initial AMX concentration = 30 mg/L, flow rate = 15 mL/s and illumination time = 90 min.

The rotational speed in SDR is one of the most efficient operation factors influencing the photocatalytic removal. This factor is dependent on the liquid film thickness and the mass transfer coefficients of pollutants^68^. The photocatalytic performance of SDR was investigated under different rotational speed (100–400 rpm) in the photodegradation of AMX as a resistant organic pollutant. The removal efficiency of AMX increases with increasing disc rotational speed and then starts to reduce when the rotational speed trespasses 300 rpm as shown in Fig. 10. Below the optimum disc rotational speed, the photodegradation efficiency of AMX is low due to the increased thickness of the liquid film on the disc surface. On the other hand, above the optimal disc rotational speed, the removal efficiency decreases due to the low retention time^68,69^. Increasing the disc rotational speed from 100 to 300 rpm resulted in an increased degradation efficiency of AMX. This trend can be explained by the following factors: (1) Turbulence and Mixing: Increasing the rotational speed enhances turbulence and mixing within the system. This increased agitation promotes better contact between the AMX molecules and the photocatalyst coated on the disc’s surface, leading to a higher interfacial area for chemical reactions to occur. (2) Mass Transfer Rate: The faster rotational speed facilitates a greater mass transfer rate. As a result, a thin sheared liquid film and very fine droplets form on the disc’s surface. This further increases the contact area and enhances the interaction between the AMX molecules and the photocatalyst^68,69^. The behavior of the liquid can be divided into two parts: before and after reaching the optimum rotational speed. Before reaching the optimum rotational speed, there is a relationship between the rotational speed and the thickness of the liquid film on the disc, where the thickness gradually decreases the rotational speed increases until it reaches an optimal thickness (at a certain speed). After surpassing the optimal rotational speed, the thickness of the liquid film decreases with increasing rotational speed, leading to a decrease in the persistence of the liquid film on the disc and consequently reducing the reaction rate. Zamani et al.^45^ and Mirzaei et al.^70^ also obtained similar results. However, it's important to note that beyond a certain point, further increments in the disc rotational speed lead to a decrease in AMX decomposition efficiency. This decrement can be attributed to the reduction in the liquid residence time on the disc. When the rotational speed becomes too high, the liquid film spends less time in contact with the photocatalyst-coated surface, limiting the opportunity for effective degradation of the AMX molecules.

#### Effect of photolysis and adsorption

**Figure 11:**
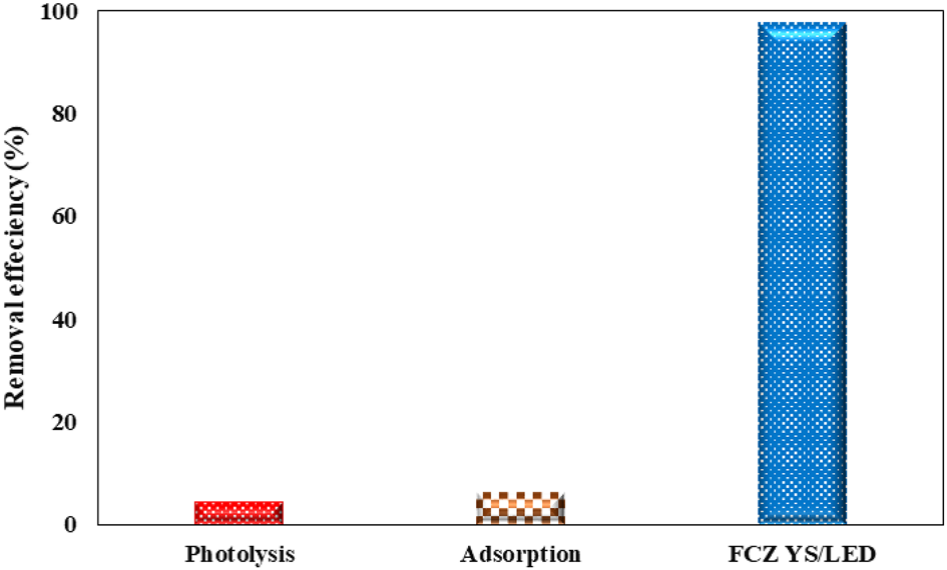
Degradation efficiency AMX for photolysis, adsorption and photocatalytic processes under optimal conditions (pH = 5, initial AMX concentration = 30 mg/L, Flow rate = 15 mL/s, Rotational speed = 300 rpm and irradiation time = 80 min).

The treatment efficiency of amoxicillin solution was evaluated by photolysis, adsorption and photocatalyst processes under optimal conditions (Fig. 11). The AMX solution was experimented without the presence of a catalyst for photolysis and without the presence of visible light for the catalyst test at 30 min. The low decomposition efficiency of AMX under LED visible light without the use of a catalyst (photolysis process) can be attributed to the lack of oxidizing species. Therefore, the effect of photolysis in the degradation of amoxicillin is negligible. The catalytic activity of the FCZ YS film without the exposure of LED visible light was weak (adsorption process) because the catalyst needs light to activate sites and generate electron-holes. But when the FCZ YS catalyst film was exposed to LED visible light (photocatalytic process), the degradation efficiency increased dramatically. The presence of light activates the electron–hole and produces free radicals and increases the degradation efficiency.

#### COD and TOC removal

**Figure 12:**
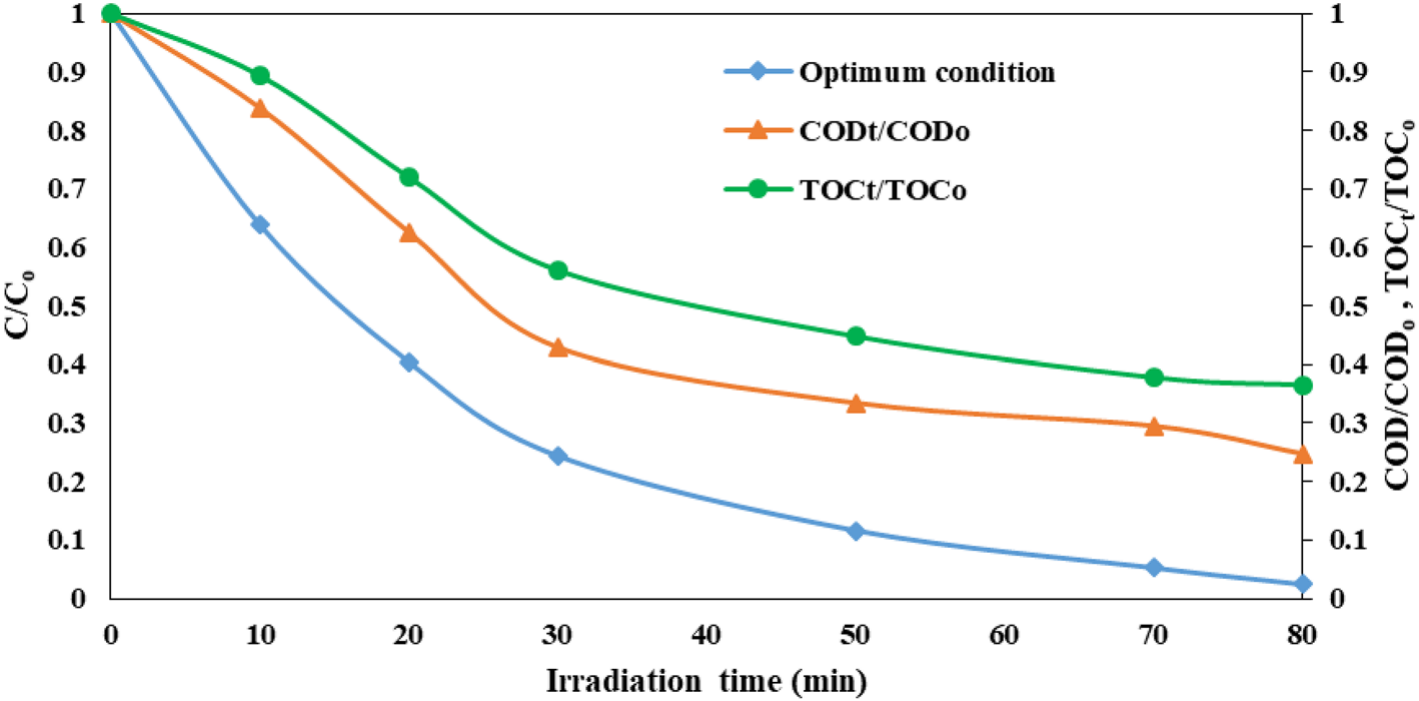
Optimal experimental conditions for COD and TOC removal.

The intermediate compounds produced by the oxidation of initial pollutants could have adverse effects on degradation efficiency due to the adsorption competition on the catalyst surface or could be higher toxicity than the original pollutant^71^. Hence, it is necessary to achieve the compounds mineralization value through the appraisal of chemical oxygen demand (COD) and total organic carbon (TOC) amounts. The change in COD and TOC during the photocatalytic process in the spinning disc reactor under the optimal conditions is exhibited in Fig. 12. The COD and TOC removal efficiency were 75.2% and 63.5% after 80 min of illumination time, respectively. At the primary of the reaction, a scarce removal of COD and TOC were seen due to the production of intermediates in organic forms that also encompass carbon. More decomposition arisen in the intermediate organic compound leads to a remarkable decrease in COD and TOC in the later stages. These results corroborate the undertaking eventuality of the FCZ YS film for pharmaceutical wastewater treatment.

#### Effect of scavengers

**Figure 13:**
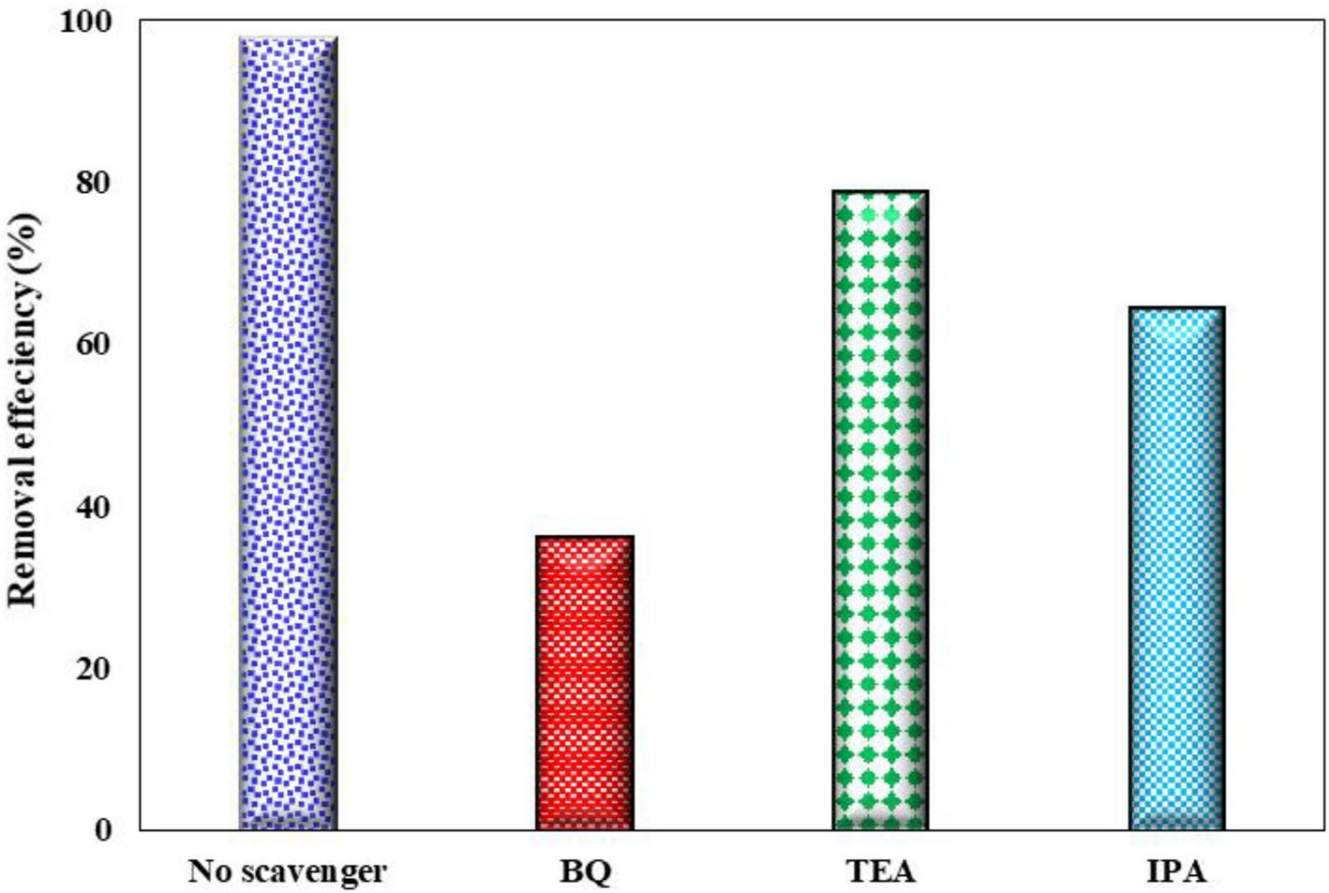
The effect of radical scavengers in photodegradation of AMX using FCZ YS film under optimal conditions (pH = 5, initial AMX concentration of 30 mg/L, Flow rate of 15 mL/s, Rotational speed of 300 rpm and irradiation time of 80 min).

To explain the photocatalytic degradation mechanism of AMX under experiment optimal conditions, the major active species produced in the photodecomposition process in SDPR were considered by evaluating the reactive oxygen species. In the active species quenching experiments, various scavengers such as triethanolamine (TEA, 1mM), benzoquinone (BQ, 1mM), and isopropanol (IPA, 1 mM) were used as an inhibitor of active holes (h^+^), superoxide radical $\left( { \cdot {\text{O}}_{{\text{2}}} ^{ - } } \right),$, and hydroxyl radical (^·^OH), respectively^72^. The results presented in Fig. 13 illustrate the distinct roles of reactive oxygen species (ROS) in the photocatalytic degradation of AMX when FCZ YS film is used. The researchers observed significant differences in the photo degradation rate of AMX upon the addition of IPA, TEA, and BQ compared to the control (no scavengers). Importantly, the addition of these scavengers had an adverse effect on the photocatalytic activity of FCZ YS film. Based on their findings, the order of reactivity for ROS on FCZ YS photocatalysts was determined as follows: BQ > IPA > TEA > no scavengers. This implies that benzoquinone (BQ) exhibited the highest reactivity towards ROS, followed isopropyl alcohol (IPA), by triethanolamine (TEA), and finally, the absence of scavengers. Consequently, it is concluded that O_2_ and OH radicals and are the main active species responsible for the photo-degradation of AMX when using FCZ YS film as the photocatalyst. These findings contribute to understanding the underlying mechanisms involved in the photocatalytic degradation process and elucidate on the role of specific ROS in the removal of AMX^73^.

#### Comparison of FCZ YS and FSCZ CS films

**Figure 14:**
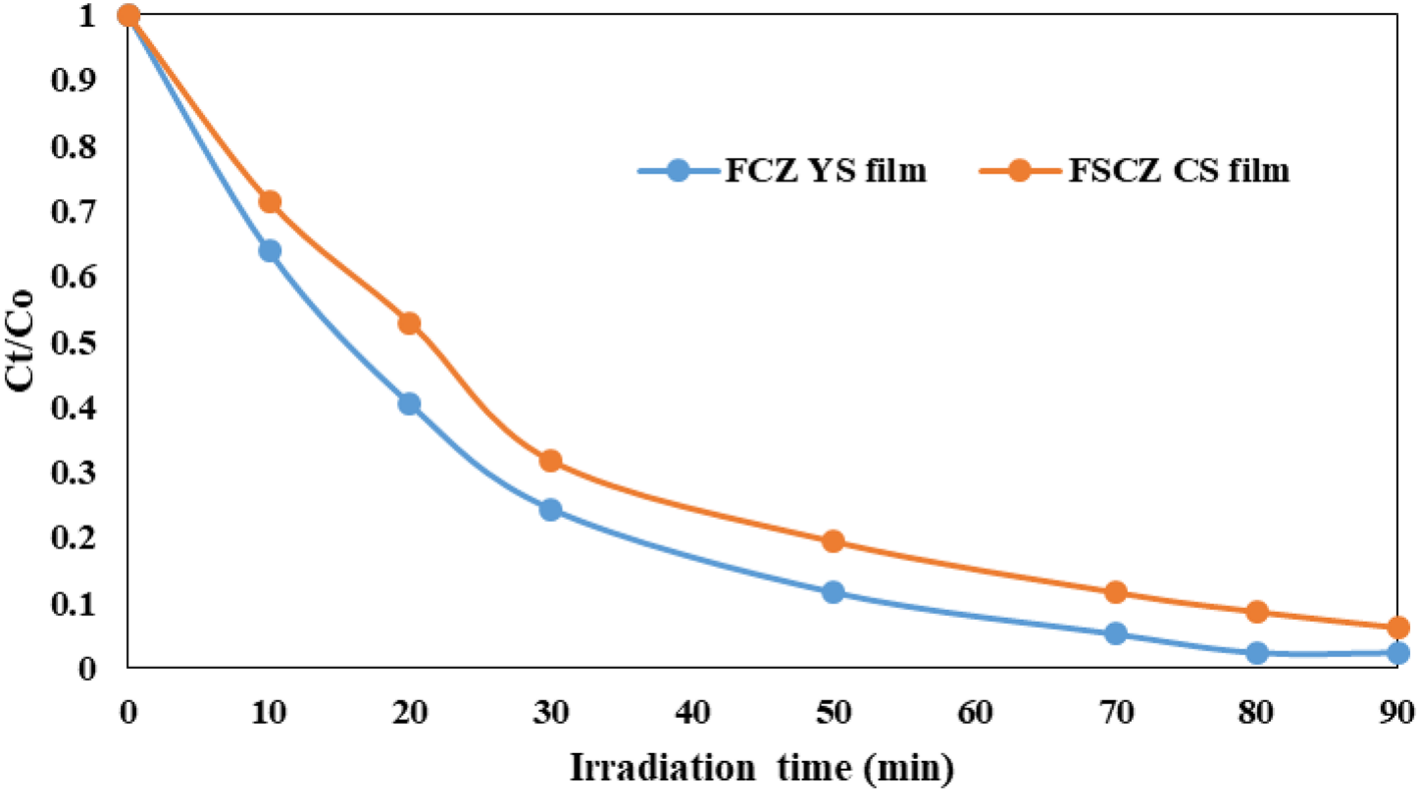
Comparison of FSCZ core–shell and FCZ yolk-shell thin films in AMX removal under optimal experimental conditions.

Figure 14 demonstrate the comparison of AMX degradation between FSCZ core–shell and FCZ yolk-shell films under optimal experimental conditions. In Fig. 14, it is observed that the FCZ yolk-shell film can degrade AMX up to 97.6% within 80 min. While, FSCZ CS film presents the degradation efficiency up to 93.75% at 90 min. This value is lower than that of the FCZ yolk-shell film. The improved catalytic performance of FCZ yolk-shell film can be attributed to their unique features. The cavity or hollow space within the yolk-shell layer acts as a nanoreactor and provides sufficient space and numerous active sites on the surface of the core and the void space between the core and the shell to carry out the reaction between AMX and the catalyst^74^. Furthermore, another reason can be attributed to the high specific surface area in the FCZ yolk-shell nanostructure compared to the FSCZ core–shell nanostructure (See section “BET analysis”). A larger surface area of the catalyst will have a higher number of active sites for adsorbing pollutant molecules. Consequently, the degradation efficiency increases, and the reaction time for the FCZ yolk-shell is reduced compared to the FSCZ core–shell film.

#### Reusability and durability of FCZ YS film experiment

**Figure 15:**
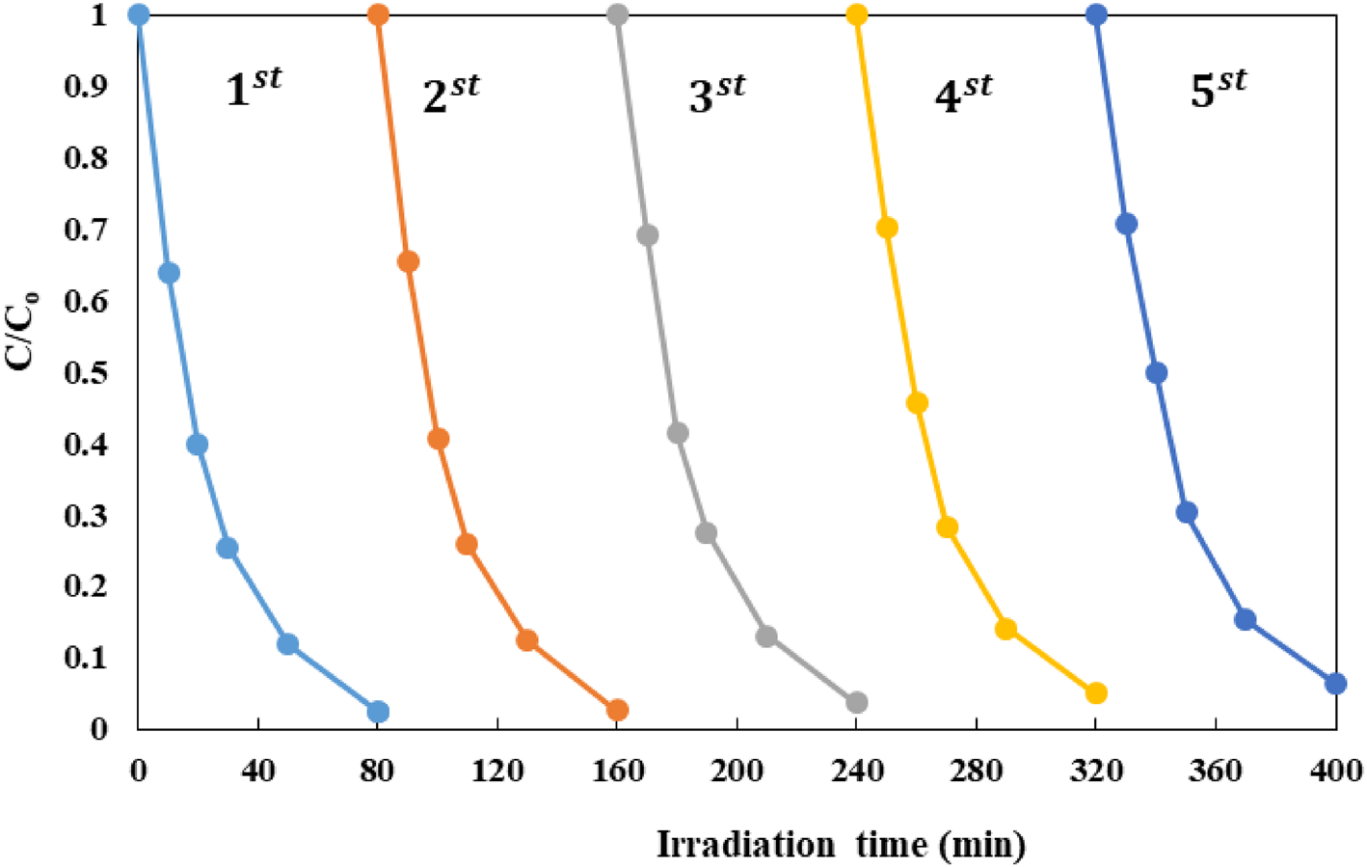
Reusability and durability of FCZ YS film as a performance of AMX degradation under optimal conditions (pH = 5, initial AMX concentration of 30 mg/L, Flow rate of 15 mL/s, Rotational speed of 300 rpm and irradiation time of 80 min).

Reusability and durability of a photocatalyst is an important factor controlling the long time performance of every photodegradation process. That's why, a study on the reusability and durability of FCZ YS film was conducted over recycling experiments. To investigate reusability and durability after each run, the FCZ YS film was taken out of the SDR and washed with DI water, and then dried at 80 °C for 3 h by the oven. The film was reused five times under the same reaction optimum operational conditions (pH = 5, initial AMX concentration of 30 mg/L, flow rate of 15 mL/s, rotational speed of 300 rpm and illumination time of 80 min) and the result is shown in Fig. 15. The results indicated that the performance of photocatalytic film remained unchanged in consecutive experiments. The FCZ YS film depicted great durability and sustained high photocatalytic activity after 5 runs, suggesting that the immobilized FCZ YS film on the ceramic disc was very durable and exhibited high potential for reusability.

## Conclusions

In this study, the prepared novel Fe_3_O_4_@void@CuO/ZnO yolk-shell catalyst were successfully deposited on the ceramic disc surface of the SDPR by the spin coating method, and applied to the photocatalytic degradation of amoxicillin pollutant in aqueous environments by LED visible light irradiation. The effects of different operational parameters (disc rotational speed, flow rate, pH, initial concentration of AMX, reaction time) on the degradation efficiency of AMX solution were investigated. The highest degradation efficiency of AMX were obtained under optimal conditions of pH = 5, the initial AMX concentration of 30 mg/L, the flow rate of 15 mL/s, rotational speed of 300 rpm and irradiation time of 80 min. Ultimately, the proper reusability and excellent durability of the prepared photocatalyst film were recorded with reproducibility of the photodegradation process for five runs at optimum conditions. According to the results obtained from the SDPR system and its excellent catalytic durability, it can be used as a proposed option on a scale-up for the treatment of pharmaceutical industry wastewaters.

### Supplementary Information

Supplementary Information.

## Data Availability

The datasets generated and analyzed during the current study available from the corresponding author on reasonable request.
