# Supplementary material for: Enhanced photocatalytic degradation of amoxicillin using a spinning disc photocatalytic reactor (SDPR) with a novel Fe3O4@void@CuO/ZnO yolk-shell thin film nanostructure
Source: Sci Rep. 2023 Sep 27;13:16185. doi: 10.1038/s41598-023-43437-8 (PMC10533499; doi:10.1038/s41598-023-43437-8)
Supplement: Supplementary file 1 — Supplementary Information. [file 41598_2023_43437_MOESM1_ESM.docx]

**Electronic Supplementary Material**

**Enhanced Photocatalytic Degradation of Amoxicillin Using a Spinning Disc Photocatalytic Reactor (SDPR) with a novel Fe_3_O_4_@void@CuO/ZnO Yolk-Shell Thin Film Nanostructure**

Saeid Fallahizadeh^a,b^, Mitra Gholami^a,b,^*, Mahmood Reza Rahimi^c,^*, Ali Esrafili^a,b^, Mahdi Farzadkia^a,b^, Majid Kermani^a,b^

a Research Center for Environmental Health Technology, Iran University of Medical Sciences, Tehran, Iran

b Department of Environmental Health Engineering, School of Public Health, Iran University of Medical Sciences, Tehran, Iran

c Process Intensification Laboratory, Department of Chemical Engineering, Yasouj University, Yasouj, 75918-74831, Iran

*Corresponding authors. E-mail address:M. Gholami (Email: [gholamim@iums.ac.ir](mailto:gholamim@iums.ac.ir)); M.R.Rahimi( mrrahimi@yu.ac.ir)


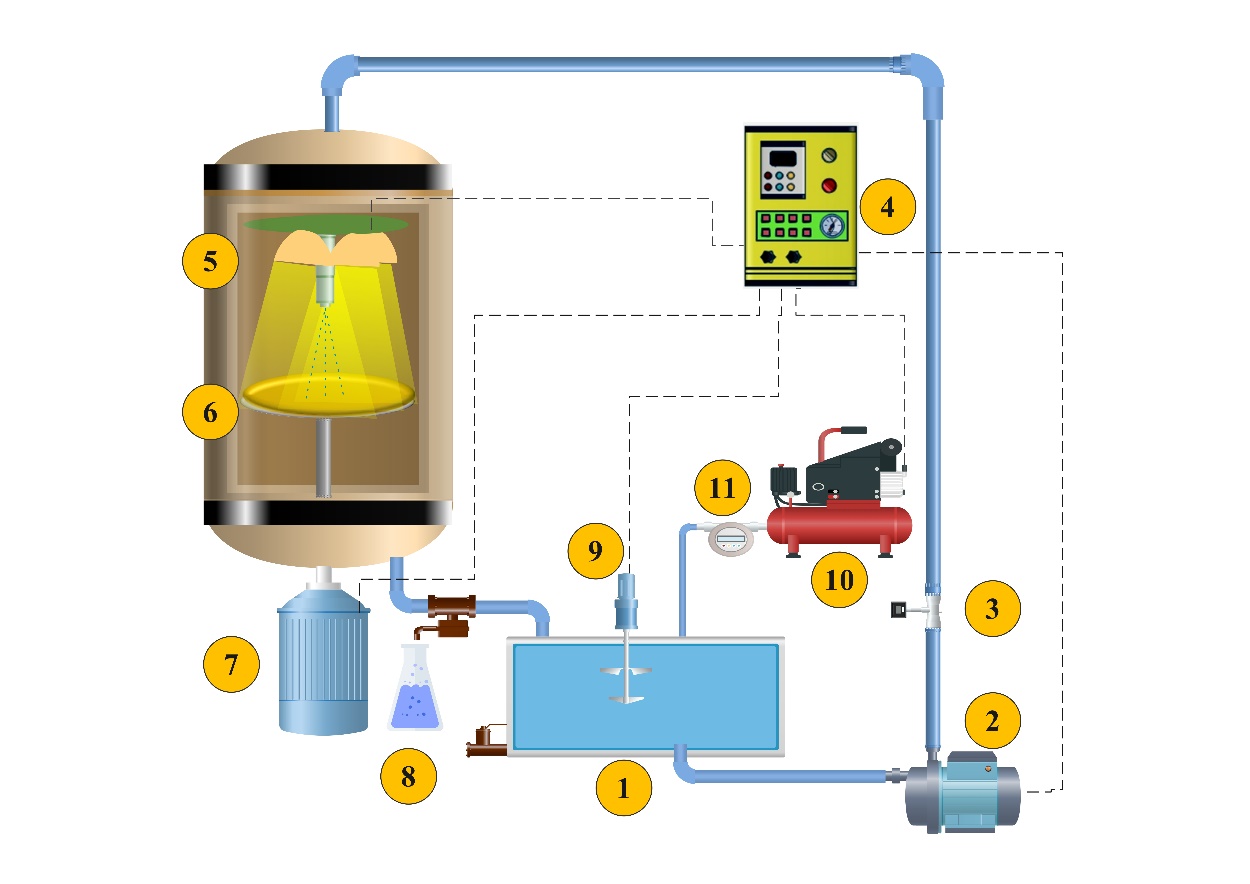


Fig. S1 Schematic details of the SDPR in this research, 1: Reservoir tank, 2: Fluid pump, 3: Flowmeter, 4: Electrical power box, 5: LED visible light source, 6: Spinning disc, 7: Motor for rotating the disc, 8: sampling location, 9: Mixer, 10: Air compressor, 11: Flowmeter. (Drawn with EdrawMax Ultimate software, version: 12.6.0.1023)

**
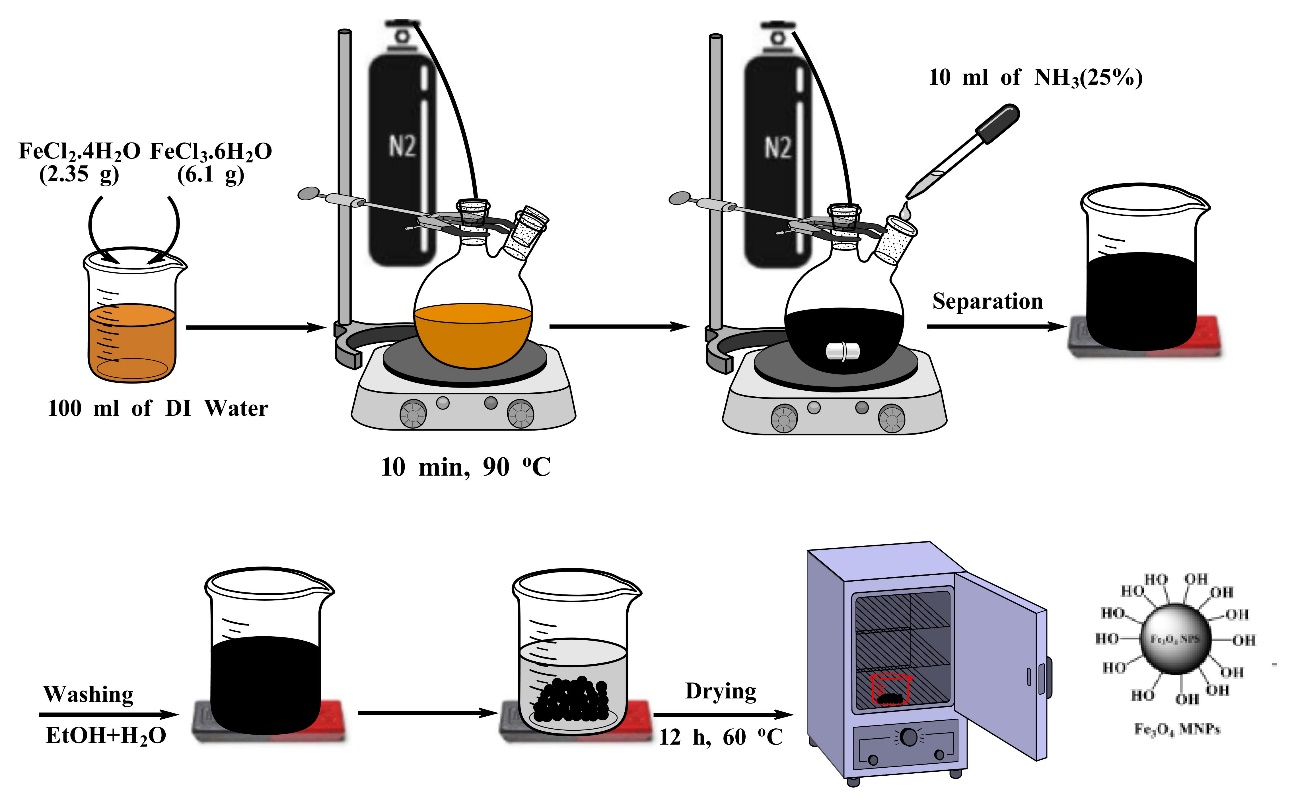
**

Fig. S2. The schematic of Fe_3_O_4_ magnetic nanoparticle synthesis (Drawn with ChemDoodle software)


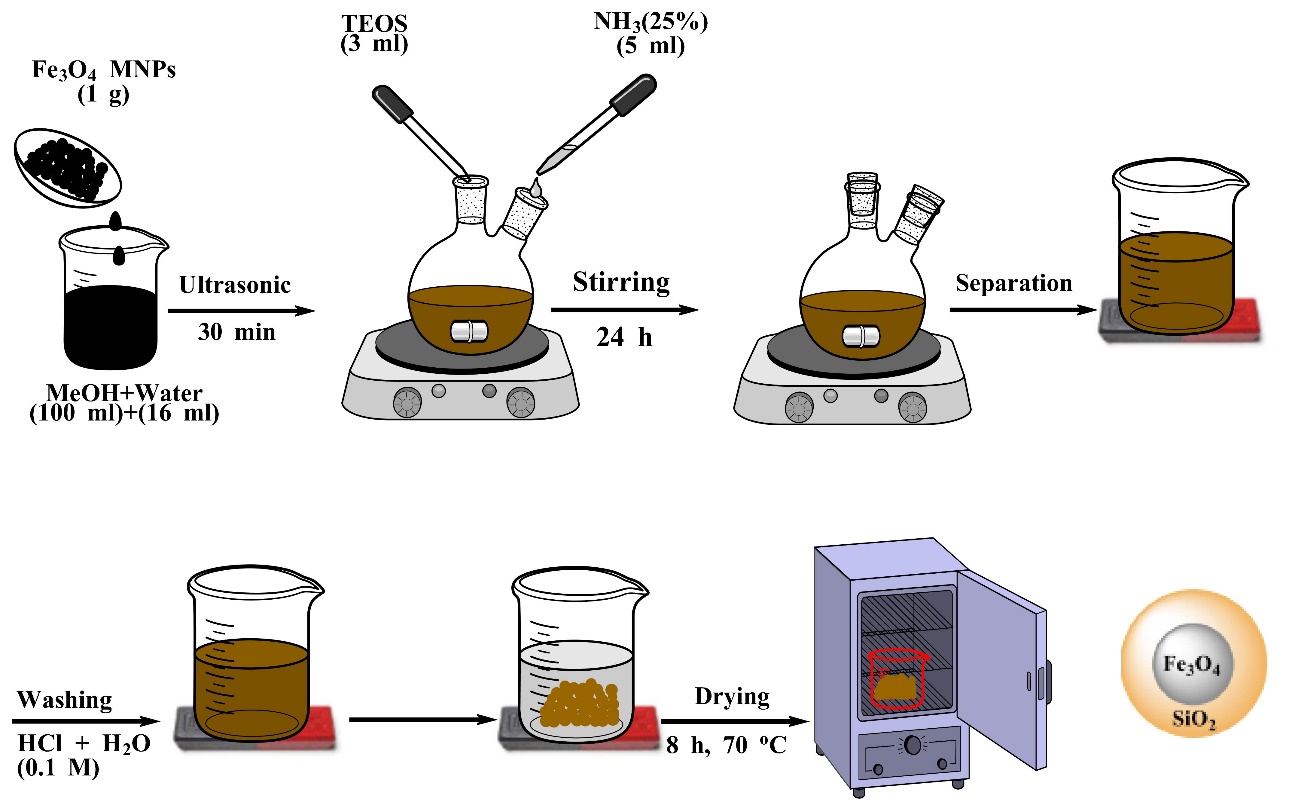


Fig. S3. The schematic of Fe_3_O_4_@SiO_2_ core-shell nanostructure synthesis (Drawn with ChemDoodle software)


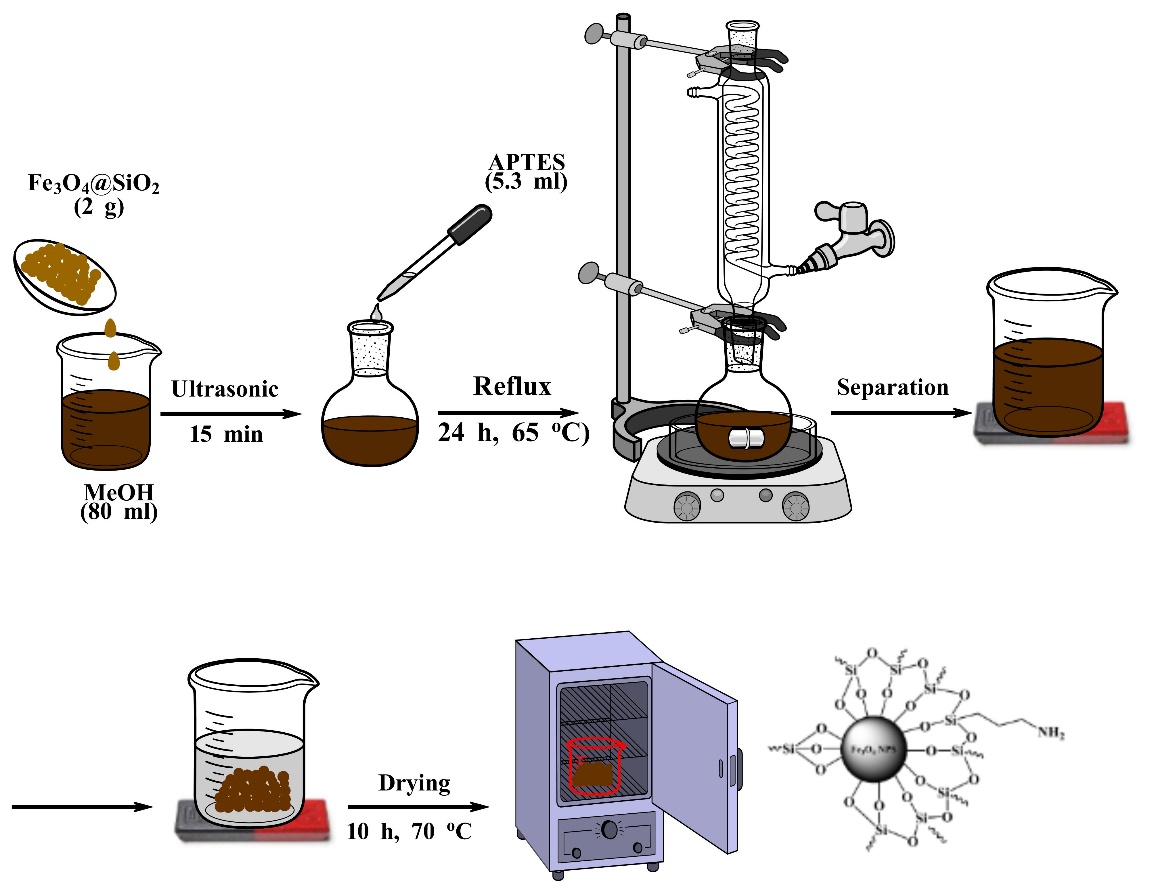


Fig. S4. The schematic of Fe_3_O_4_@SiO_2_-NH_2_ core-shell nanostructure synthesis (Drawn with ChemDoodle software)


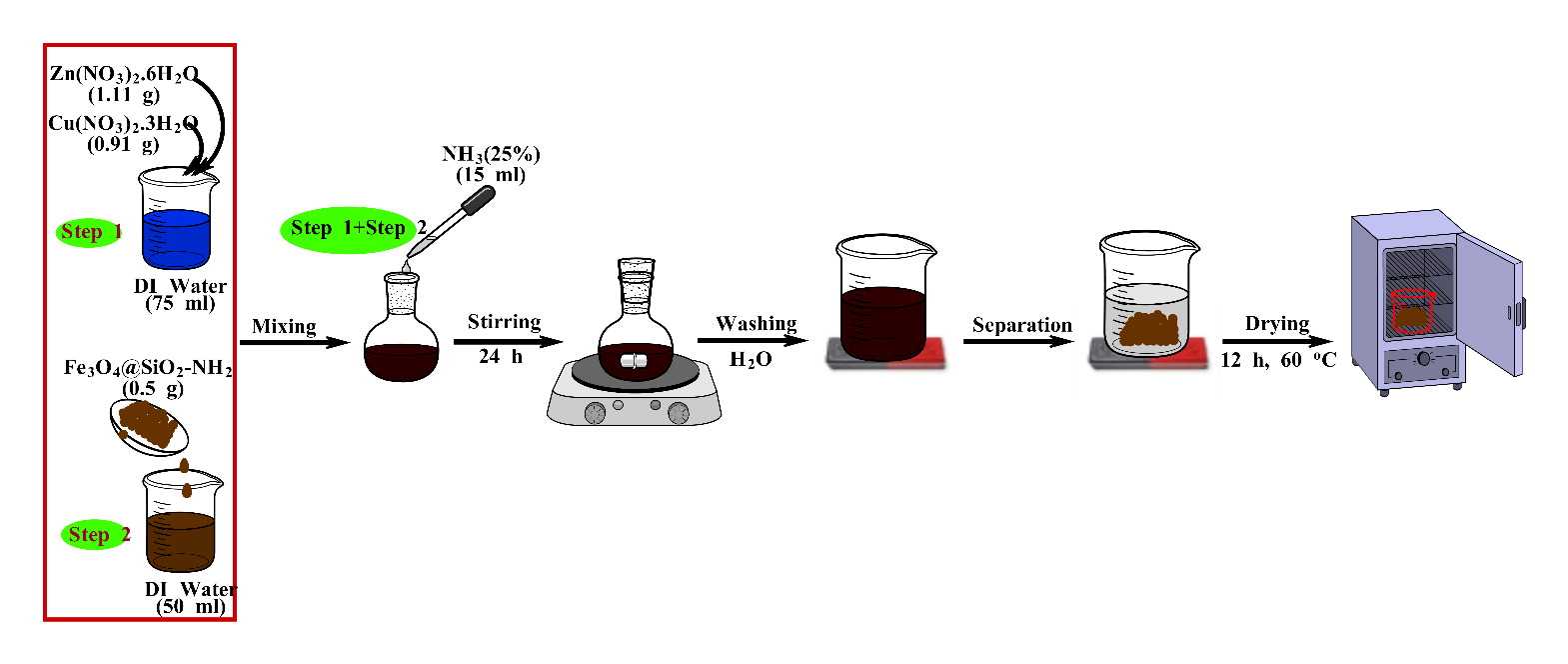


Fig. S5. The schematic of Fe_3_O_4_@SiO_2_-NH_2_@CuO-ZnO core-shell nanostructure synthesis (Drawn with ChemDoodle software)


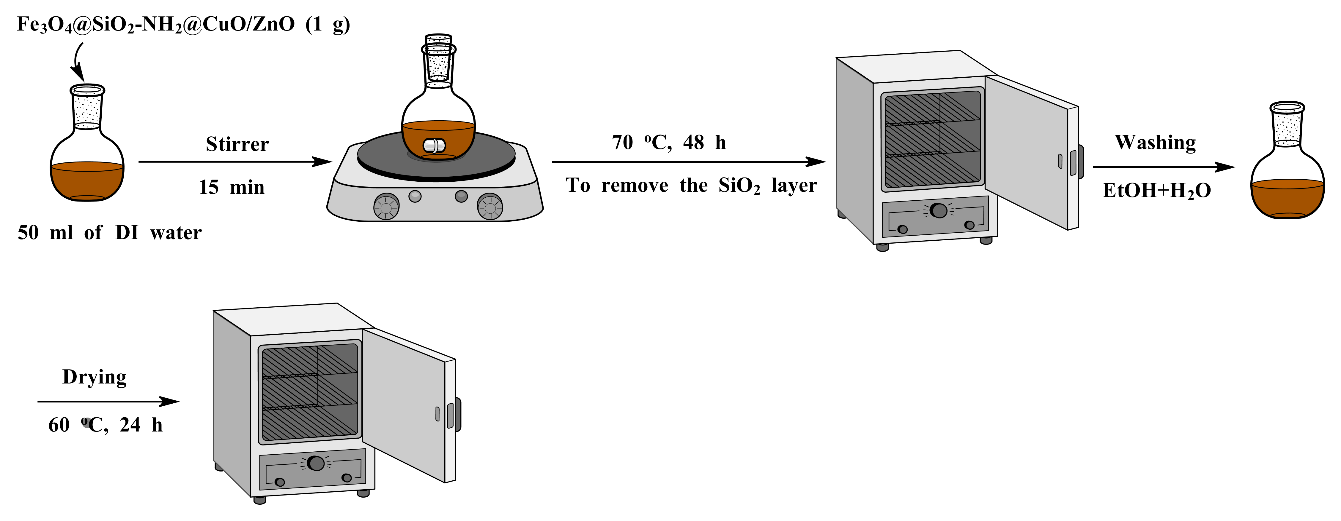


Fig. S6. The schematic of Fe_3_O_4_@Void@CuO-ZnO yolk-shell nanostructure synthesis (Drawn with ChemDoodle software)


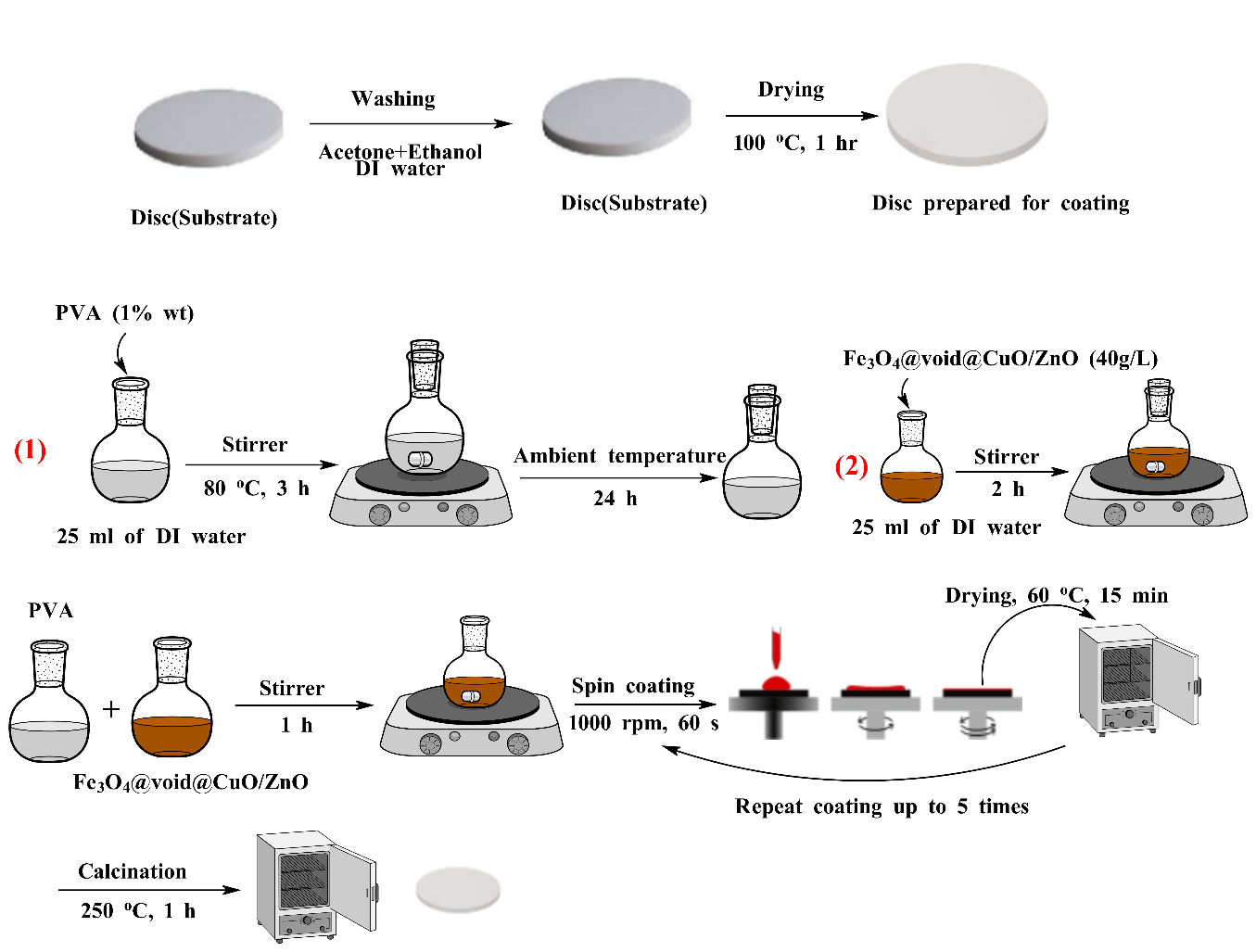


Fig. S7. The schematic of the deposition of Fe_3_O_4_@Void@CuO-ZnO yolk-shell nanostructure thin film on the ceramic disc (Drawn with ChemDoodle software)


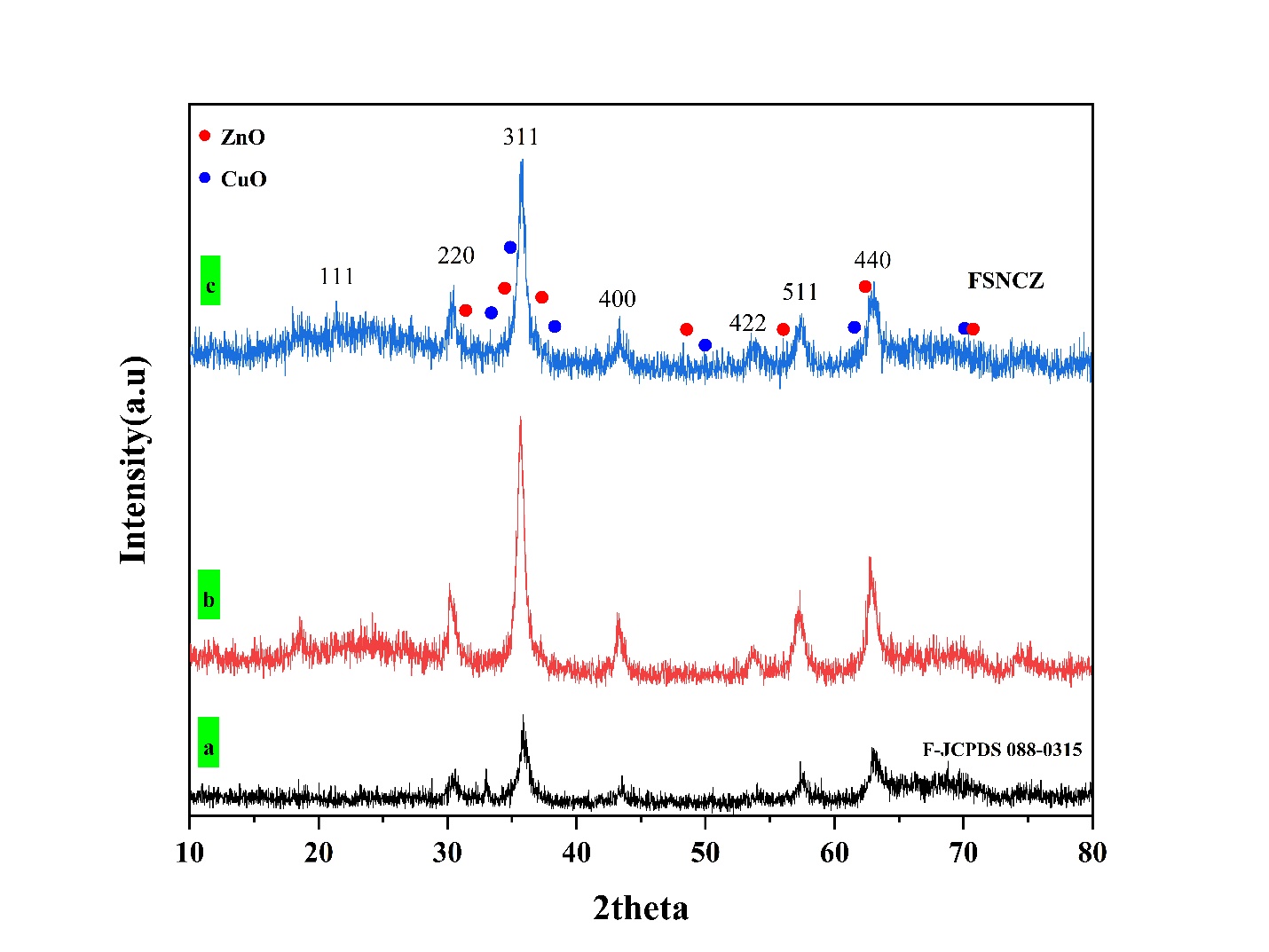


Fig. S8.The XRD patterns of pure Fe_3_O_4_ MNP (a), Fe_3_O_4_@SiO_2_ core-shell (b) and Fe_3_O_4_@SiO_2_@CuO/ZnO(c) core-shell nanostructures


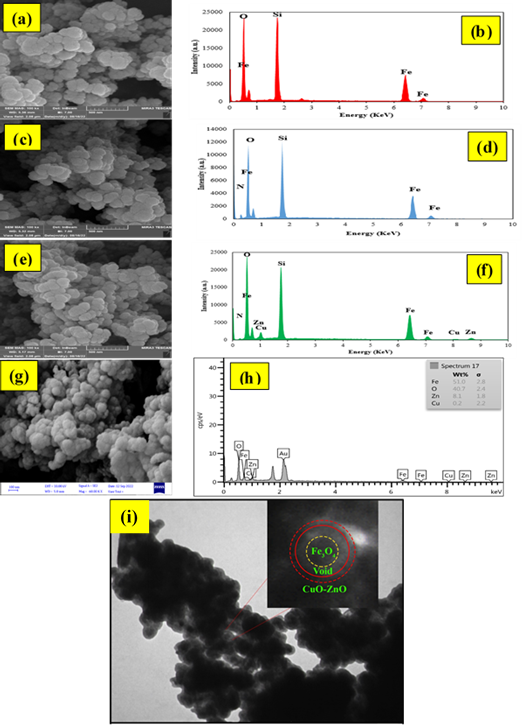


Fig. S9 FE-SEM and EDX images of (a-b) Fe_3_O_4_@SiO_2_, (c-d) Fe_3_O_4_@SiO_2_-NH_2_, (e-f) Fe_3_O_4_@SiO_2_@CuO/ZnO Core-Shell, (g-h) Fe_3_O_4_@void@CuO/ZnO Yolk-Shell and (i) TEM image of Fe_3_O_4_@void@CuO/ZnO Yolk-Shell.

Table S1 the ratio of specific surface area in different core-shell and yolk-shell composites

| Core-shell  Surface area (m^2^/g) | Yolk-shell  Surface area (m^2^/g) | Specific surface area ratio (YS/CS) | References |
| --- | --- | --- | --- |
| Fe_3_O_4_@SiO_2_-pd@mCeO_2_  95 m^2^/g | Fe_3_O_4_@h-pd@mCeO_2_  90 m^2^/g | 0.95 | [1] |
| Fe_3_O_4_@SiO_2_@CdS  25.1 m^2^/g | Fe_3_O_4_@Void@CdS  32.9 m^2^/g | 1.31 | [2] |
| Fe_3_O_4_@TNS  114.9 m^2^/g | Fe_3_O_4_@TNS  130.1 m^2^/g | 1.13 | [3] |
| Fe_3_O_4_@SiO_2_  714 m^2^/g | Fe_3_O_4_@SiO_2_  592 m^2^/g | 0.83 | [4] |
| Au@TiO_2_  34.1 m^2^/g | Au@TiO_2_  128.9 m^2^/g | 3.78 | [5] |
| Fe_3_O_4_@SiO_2_@CuO-ZnO  18.98 m^2^/g | Fe_3_O_4_@Void@CuO-ZnO  62.48 m^2^/g | 3.29 | This work |

Table. S2 Specific surface area and pore parameters of FSCZ CS and FCZ YS photocatalyst film

| Photocatalyst | SBET (m^2^/g) | Pore volume (cm^3^/g) | Pore diameter (nm) |
| --- | --- | --- | --- |
| FSCZ CS | 18.98 | 0.0053 | 4.62 |
| FCZ YS | 62.48 | 0.057 | 5.94 |

**References**

1. Li, Y., et al., *Magnetic Core–Shell to Yolk–Shell Structures in Palladium‐Catalyzed Suzuki–Miyaura Reactions: Heterogeneous versus Homogeneous Nature.* ChemPlusChem, 2016. **81**(6): p. 564-573.

2. Shi, W., et al., *Synthesis of yolk–shell structured Fe3O4@ void@ CdS nanoparticles: a general and effective structure design for photo-fenton reaction.* ACS Applied Materials & Interfaces, 2016. **8**(32): p. 20831-20838.

3. Yin, L., et al., *Rationally designed core-shell and yolk-shell magnetic titanate nanosheets for efficient U (VI) adsorption performance.* Environmental Pollution, 2018. **238**: p. 725-738.

4. Yang, Y., et al., *Organosilane-assisted transformation from core–shell to yolk–shell nanocomposites.* Chemistry of Materials, 2011. **23**(16): p. 3676-3684.

5. Wang, Y., et al., *Influence of yolk-shell Au@ TiO2 structure induced photocatalytic activity towards gaseous pollutant degradation under visible light.* Applied Catalysis B: Environmental, 2019. **251**: p. 57-65.
